# The Effect of Abiotic Stress Conditions on Expression of Calmodulin (*CaM*) and Calmodulin-Like (*CML*) Genes in Wild-Growing Grapevine *Vitis amurensis*

**DOI:** 10.3390/plants8120602

**Published:** 2019-12-13

**Authors:** Alexandra S. Dubrovina, Olga A. Aleynova, Zlata V. Ogneva, Andrey R. Suprun, Alexey A. Ananev, Konstantin V. Kiselev

**Affiliations:** 1 Laboratory of Biotechnology, Federal Scientific Center of the East Asia Terrestrial Biodiversity, Far Eastern Branch of the Russian Academy of Sciences, 690022 Vladivostok, Russiakiselev@biosoil.ru (K.V.K.); 2 Department of Biodiversity, The School of Natural Sciences, Far Eastern Federal University, 690090 Vladivostok, Russia

**Keywords:** calmodulin (CaM), calmodulin-like proteins (CML), abiotic stress, grapevine, gene expression

## Abstract

Plant calmodulins (CaMs) and calmodulin-like proteins (CMLs) are important plant Ca^2+^-binding proteins that sense and decode changes in the intracellular Ca^2+^ concentration arising in response to environmental stimuli. Protein Ca^2+^ sensors are presented by complex gene families in plants and perform diverse biological functions. In this study, we cloned, sequenced, and characterized three *CaM* and 54 *CML* mRNA transcripts of *Vitis amurensis* Rupr., a wild-growing grapevine with a remarkable stress tolerance. Using real-time quantitative RT-PCR, we analyzed transcript abundance of the identified *VaCaMs* and *VaCMLs* in response to water deficit, high salinity, high mannitol, cold and heat stresses. Expression of *VaCaMs* and 32 *VaCMLs* actively responded to the abiotic stresses and exhibited both positive and negative regulation patterns. Other *VaCML* members showed slight transcriptional regulation, remained essentially unresponsive or responded only after one time interval of the treatments. The substantial alterations in the *VaCaM* and *VaCML* transcript levels revealed their involvement in the adaptation of wild-growing grapevine to environmental stresses.

## 1. Introduction

Plants, as sessile organisms, have to develop multiple biochemical and physiological reactions in order to adapt to the constantly changing environmental conditions. Different abiotic stress stimuli, including unfavorable temperatures, drought, flooding, or soil salinity, affect plant growth and productivity. These and other abiotic stresses evoke spatially and temporally distinct alterations in the intracellular Ca^2+^ concentrations, i.e., “Ca^2+^ signatures”, which are perceived and decoded by Ca^2+^ binding proteins referred to as Ca^2+^ sensors [1,2]. The majority of Ca^2+^ sensor proteins contain several EF-hand motifs, conserved helix-loop-helix structures, in which the Ca^2+^ ions are coordinated within the acidic Ca^2+^-coordinating loop [3]. Furthermore, recent studies indicated that Ca^2+^ signals are implicated in the signal transmission at long distances or even at the organismic level [4,5]. The major plant EF-hand-containing Ca^2+^-binding proteins are divided into calmodulins (CaMs), calmodulin-like proteins (CML), Ca^2+^-dependent protein kinases (CDPKs), and calcineurin B-like proteins (CBLs) [1,6,7].

CaM is a functionally important and highly conserved Ca^2+^ sensor present in all eukaryotic organisms, while CMLs are Ca^2+^ sensors closely related to CaMs but present only in higher plants [8,9]. CaMs are small proteins containing four EF-hands; CMLs greatly vary in their length and EF-hand number containing from one to six EF-hand motifs. Plant CaMs and CMLs do not exhibit catalytic activity and act as sensor relays that transmit the information encoded by the Ca^2+^ signatures to downstream events, such as protein interaction, protein phosphorylation, metabolic changes, or gene regulation [8,10].

Plant CaMs and CMLs are known to function in plant developmental processes and in response to both biotic and abiotic stresses [2,11,12,13]. It is known that gene expression levels of plant *CaMs* and *CMLs* are regulated by a variety of abiotic stress stimuli, e.g., in tea, apple and soybean in response to cold, drought, flooding, or high salinity [14,15,16]. Transgenic plants overexpressing certain stress-responsive *CaMs* and *CMLs* showed greater tolerance to the respective or even multiple abiotic stress treatments [17,18,19,20] or, in turn, were rendered more sensitive to the applied abiotic stresses [21]. There is also evidence that some plant CaMs and CMLs interact with the protein targets that are known to regulate abiotic stress adaptation either positively or negatively [22,23,24].

A recent study has identified and characterized *CaM* and *CML* gene families in cultured grapevine *Vitis vinifera* L. based on the genome sequencing data and publicly available expression profiling datasets [25]. The purpose of the present study was to identify the *CaM* and *CML* genes actively expressed in wild-growing grape *Vitis amurensis* Rupr. in response to various abiotic stress stimuli and analyze their expression under these stress conditions. Wild-growing relatives of cultured plant species often exhibit higher tolerance to abiotic stresses, and genes of wild plant species represent an important source for improving abiotic stress tolerance of cultivated counterparts.

## 2. Results

### 2.1. Isolation, Molecular Cloning, and Sequencing of VaCaM and VaCML Transcripts

To clone and sequence the full-length coding sequences of *CaMs* and *CMLs* of *V. amurensis*, we designed specific primers to the 5′ and 3′ ends of the highly homologous *CaMs* and *CMLs* of *V. vinifera* (Table 1; Table S1). *V. vinifera* is a close species to *V. amurensis*, and its genome was sequenced [26,27]. To design the specific primers, we retrieved the predicted *CaMs* and *CMLs* of *V. vinifera* PN20024 genotype (V2 proteome prediction) present in the Grapevine Genome Centro Di Ricerca Interdipartimentale Per Le Biotecnologie Innovative (CRIBI) Biotech Centre database using the CaM and CML protein sequences of *A. thaliana* downloaded from the Arabidopsis Information Resource (TAIR) database as queries as described [25]. In addition, we downloaded all CaM and CML protein sequences of *V. vinifera* predicted by an automated computational analysis and deposited to the National Centre for Biotechnology Information (NCBI) GenBank. After the removal of all duplicate sequences and sequences containing functional domains other than EF-hands, we obtained a total of three *VviCaMs* and 68 *VviCMLs*.

**Figure 1:**
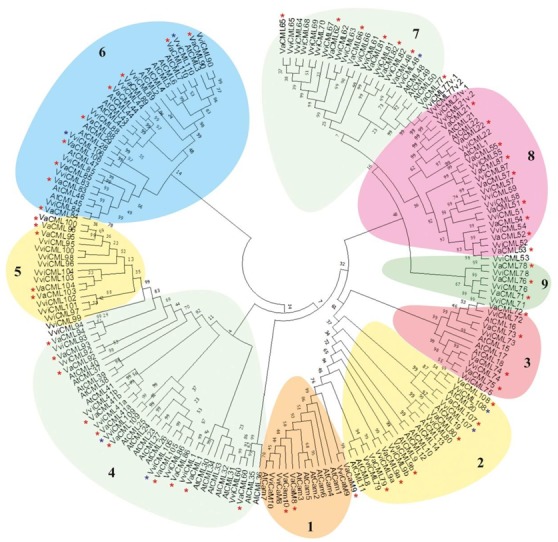
Phylogenetic tree of plant calmodulins (CaMs) and calmodulin-like proteins (CMLs) of *Vitis amurensis*, *Vitis vinifera*, and *Arabidopsis thaliana* created using the full-length protein sequences. The phylogenetic tree was constructed using the MEGA-X program by the neighbor joining method with 1000 bootstrap replicates. The CaMs and CMLs were categorized into nine subgroups highlighted with different colors. Red asterisks denote CaMs and CMLs of *V. amurensis* identified in this study. Dark blue asterisks denote CaMs and CMLs of *V. vinifera* firstly described in this study.

We compared our results with the computational analysis of *CaMs* and *CMLs* of *V. vinifera* published by Vandelle et al. [25]. In addition to the three *VviCaMs* and 62 *VviCMLs* described previously for *V. vinifera*, our analysis of the *V. vinifera CaMs* and *CMLs* sequences available at the CRIBI and NCBI databases identified seven additional *VviCMLs*, including *VviCML48*, *VviCML105*, *VviCML106*, *VviCML107*, *VviCML108*, *VviCML109*, and *VviCML110* (Table 1; Figure 1). The seven newly identified *VviCMLs* were named according to the procedure described by Vandelle et al. [25] following the instructions of the international Super-Nomenclature Committee for Grape Gene Annotation [28]. The deduced amino acid sequences of the newly identified *VviCMLs* did not contain functional domains other than EF-hands (Table 1). We also noted that nucleotide sequences of the previously identified *VviCML90* and *VviCML91* shared 100% identity with each other at the nucleotide level, and they should be treated as one *VviCML*.

Using RT-PCRs with the primers designed to the three *VviCaMs* and 68 *VviCMLs* (Table S1), we cloned and sequenced cDNAs containing full-length coding DNA sequences (CDS) of *CaM* and *CML* transcript variants from wild grape *V. amurensis* using RNA isolated from unstressed leaves and stems of *V. amurensis*. The analysis identified three *VaCaMs* and 54 *VaCMLs* expressed in the cuttings of *V. amurensis*. Table 1 shows a comparison of the cloned and sequenced *VaCaMs* and *VaCMLs* with those identified for *V. vinifera*. The deduced amino acid sequences of the cloned and sequenced *VaCaMs* and *VaCMLs* shared high identities (95–100%) with the homologous *CaMs* and *CMLs* of *V. vinifera* predicted by the automated computational analysis (Table 1).

A phylogenetic analysis was performed on the full-length amino acid sequences of the CaMs and CMLs from *V. vinifera*, *V. amurensis*, and *A. thaliana* (Figure 1). The proteins were categorized into nine subgroups based on sequence similarities and relationships of CaMs and CMLs of the analyzed plant species and taking into attention the described classification for *V. vinifera* proteins [25]. The deduced amino acid sequences of the newly identified *VviCMLs* clustered into the subgroup 2 (VviCML107, VviCML108), subgroup 4 (VviCML105, VviCML109), subgroup 6 (VviCML106, VviCML110), and subgroup 7 (*VviCML48*). The newly identified VviCMLs possessed from two to four putative EF-hand motifs (Table 1).

The identified three *VaCaMs* and 54 *VaCMLs* shared a common structure with other plant and *V. vinifera CaMs* and *CMLs* and possessed from one to five putative EF-hand motifs, except for *VaCML71*. The deduced amino acid sequence of *VaCML71* did not contain any putative EF-hand motifs due to the presence of a premature stop codon after a 141 bp insertion, which resembled intron retention due to the presence of canonical 5′ and 3′ splice sites. Notably, the *VaCML86* transcript contained a 210-bp insertion (no frameshift) and contained a higher number of putative EF-hands in comparison with the homologous *VviCML86*. Most of the VaCMLs were predicted to be myristoylated or palmitoylated proteins (Table 1), which indicates possible protein-membrane interactions of these CMLs.

### 2.2. Expression of VaCaMs and VaCMLs in Response to Abiotic Stress Conditions

To test the involvement of *VaCaM* and *VaCML* genes in the responses of *V. amurensis* to the water deficit, high salt, high mannitol, cold, and heat abiotic stress conditions, we used the *V. amurensis* cuttings (excised young stems with one healthy leaf) for the control non-stress and abiotic stress treatments. Total RNA was isolated from the leaves of the treated cuttings of *V. amurensis* 6 h, 12 h, and 24 h post-treatments. The *V. amurensis* cuttings were placed in filtered water at +25 °C (non-stress treatment), on a paper towel at +25 °C (desiccation or water deficit stress), in 0.4 M NaCl at +25 °C (high salt stress) and 0.4 M mannitol at +25 °C (high mannitol stress). To apply cold and heat temperature stress, the cuttings were incubated in filtered water in a growth chamber at +4 °C, +10 °C, and +37 °C. We used a similar experimental design and chemical concentrations as in Chung et al. [30] and Dubrovina et al. [33] for studying *CDPK* gene expression in *Capsicum annuum* and *V. amurensis*, respectively. Then, we applied qRT-PCR for the analysis of *VaCaM* and *VaCML* gene expression.

**Figure 2:**
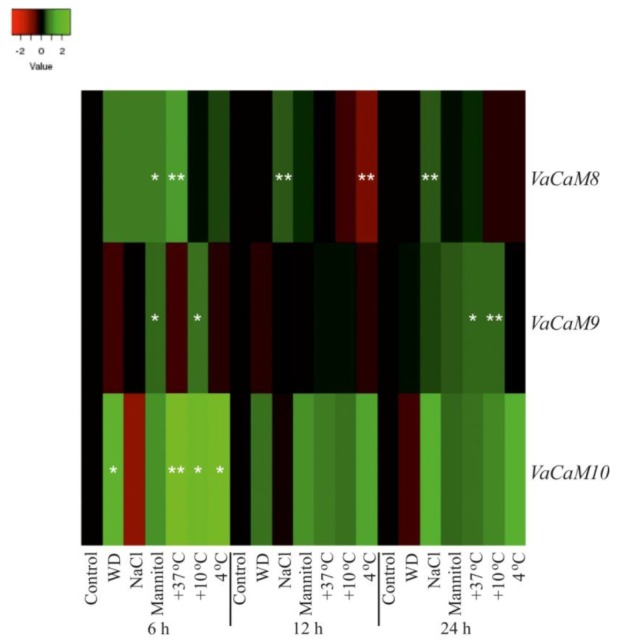
Heatmap of *VaCaM* expression levels after 6 h, 12 h, and 24 h of treatments in *Vitis amurensis* cuttings exposed to abiotic stress conditions. The *VaCaM* expression levels were determined by quantitative RT-PCR. The color scale represents increased (green) and decreased (red) log_2_ fold changes of the expression values under abiotic stress treatments relative to the control. Control—non-stress conditions (filtered water, +25 °C); WD—water-deficit stress (cuttings laid on a paper towel, +25 °C); NaCl—salt stress (0.4 M NaCl, +25 °C); Mannitol—osmoticum (0.4 M mannitol, +25 °C); +37 °C—heat stress (filtered water, +37 °C); +10 °C, and +4 °C—cold stress (filtered water, +10 °C, and +4 °C). *, **—significantly different from the values of *CaM* expression in *V. amurensis* under the control conditions after 6 h, 12 h, or 24 h of treatments at *p* ≤ 0.05 and 0.01 according to the Student’s *t*-test.

The qRT-PCR data revealed that *VaCaM8* transcript levels considerably increased under the high salt stress conditions in 1.4–1.8 times at all time intervals post-treatment (Figure 2 and Figure S1). Also, the *VaCaM8* expression was affected under +37 °C, high mannitol, and +4 °C stress conditions but only at one time interval. The *VaCaM9* gene was responsive to low temperature stress (+10 °C), with considerable increases in transcript levels detected after 6 h and 24 h of treatment (Figure 2 and Figure S1). However, incubation at +4 °C did not cause such elevation to the *VaCaM9* expression. Transcript levels of *VaCaM9* also responded to mannitol and heat but at one time interval after being exposed to the stresses (Figure 2 and Figure S1). The *VaCaM10* gene responded to the abiotic stresses more actively than *VaCaM8* and *VaCaM9*, but the detected alterations were considerable only after 6 h of treatments (Figure 2 and Figure S1).

**Figure 3:**
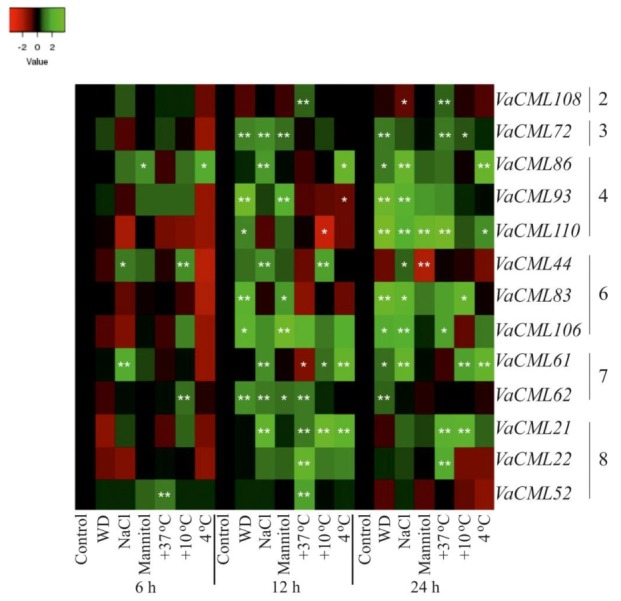
Heatmap of expression levels of 13 *VaCMLs* significantly up-regulated at least at two time intervals after one or several abiotic stress treatments in *Vitis amurensis* cuttings. The *VaCaM* expression levels were determined by quantitative RT-PCR and analyzed after 6 h, 12 h, and 24 h of treatments. The color scale represents increased (green) and decreased (red) log_2_ fold changes of the expression values under abiotic stress treatments relative to the control. The *VaCML* genes were ordered by their subgroup numbers. Control—non-stress conditions (filtered water, +25 °C); WD—water-deficit stress (cuttings laid on a paper towel, +25 °C); NaCl—salt stress (0.4 M NaCl, +25 °C); Mannitol—osmoticum (0.4 M mannitol, +25 °C); +37 °C—heat stress (filtered water, +37 °C); +10 °C, and +4 °C—cold stress (filtered water, +10 °C, and +4 °C). *, **—significantly different from the values of *CML* expression in *V. amurensis* under the control conditions after 6 h, 12 h, or 24 h of treatments at *p* ≤ 0.05 and 0.01 according to the Student’s *t*-test.

**Figure 4:**
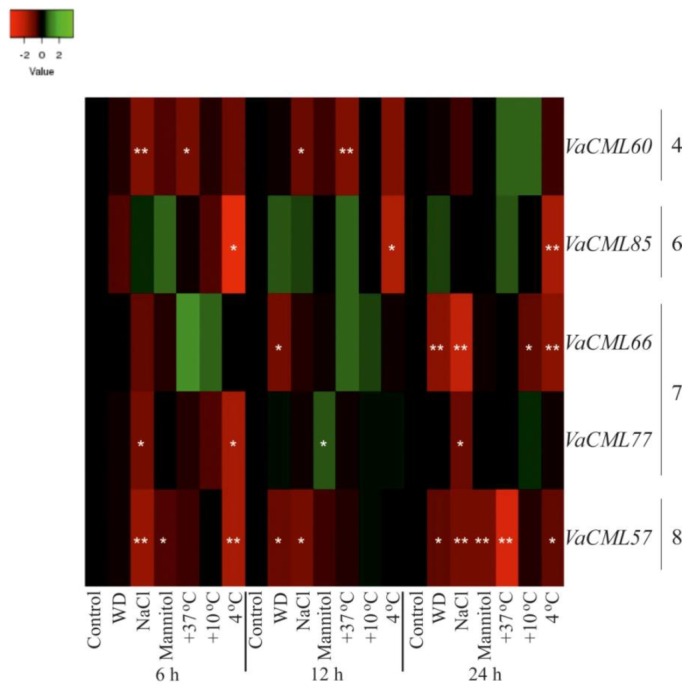
Heatmap of expression levels of five *VaCMLs* significantly down-regulated at least at two time intervals after one or several abiotic stress treatments in *Vitis amurensis* cuttings. The *VaCaM* expression levels were determined by quantitative RT-PCR and analyzed after 6 h, 12 h, and 24 h of treatments. The color scale represents increased (green) and decreased (red) log_2_ fold changes of the expression values under abiotic stress treatments relative to the control. The *VaCML* genes were ordered by their subgroup numbers. Control—non-stress conditions (filtered water, +25 °C); WD—water-deficit stress (cuttings laid on a paper towel, +25 °C); NaCl—salt stress (0.4 M NaCl, +25 °C); Mannitol—osmoticum (0.4 M mannitol, +25 °C); +37 °C—heat stress (filtered water, +37 °C); +10 °C and +4 °C—cold stress (filtered water, +10 °C, and +4 °C). *, **—significantly different from the values of *CML* expression in *V. amurensis* under the control conditions after 6 h, 12 h, or 24 h of treatments at *p* ≤ 0.05 and 0.01 according to the Student’s *t*-test.

**Figure 5:**
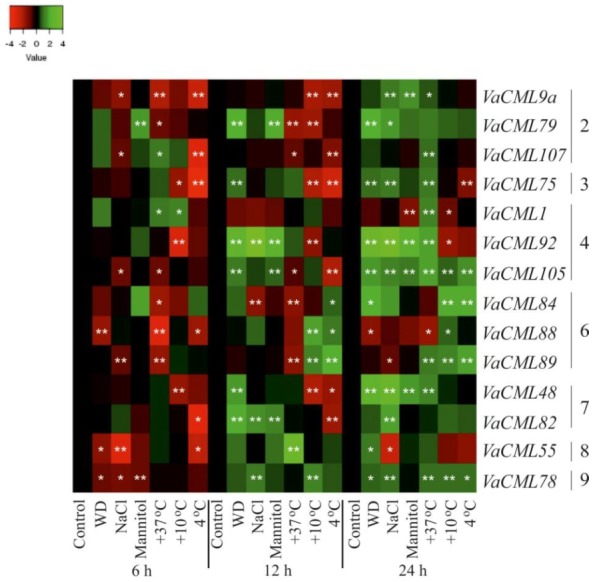
Heatmap of expression levels of 14 *VaCMLs* displaying both up- and down-regulation at least at two time intervals after one or several abiotic stress treatments in *Vitis amurensis* cuttings. The *VaCaM* expression levels were determined by quantitative RT-PCR and analyzed after 6 h, 12 h, and 24 h of treatments. The color scale represents increased (green) and decreased (red) log_2_ fold changes of the expression values under abiotic stress treatments relative to the control. The *VaCML* genes were ordered by their subgroup numbers. Control—non-stress conditions (filtered water, +25 °C); WD—water-deficit stress (cuttings laid on a paper towel, +25 °C); NaCl—salt stress (0.4 M NaCl, +25 °C); Mannitol—osmoticum (0.4 M mannitol, +25 °C); +37 °C—heat stress (filtered water, +37 °C); +10 °C and +4 °C—cold stress (filtered water, +10 °C and +4 °C). *, **—significantly different from the values of *CML* expression in *V. amurensis* under the control conditions after 6 h, 12 h, or 24 h of treatments at *p* ≤ 0.05 and 0.01 according to the Student’s *t*-test.

Then, we analyzed transcript levels of the 54 identified *VaCMLs* in response to desiccation, high salt, high mannitol, cold, and heat stresses (Figure 3, Figure 4, Figure 5 and Figures S1–S6). The *VaCMLs* were divided into four groups based on their responsiveness to the abiotic stresses:

(1) Thirteen *VaCML* genes were significantly up-regulated at a minimum of two time intervals after one or several abiotic stress treatments (Figure 3 and Figure S2);
(2) Five *VaCML* genes were significantly down-regulated at a minimum of two time intervals after one or several abiotic stress treatments (Figure 4 and Figure S3);
(3) Fourteen *VaCML* genes were differentially regulated displaying both up- and down-regulation at a minimum of two time intervals post-treatment (Figure 5 and Figure S4);
(4) Expression levels of 22 *VaCML* genes showed slight effects or occasional regulation mainly at one time interval (Figure S5) or were not essentially changed (Figure S6).

Notably, expression levels of *VaCML95*, *VaCML96*, *VaCML100*, *VaCML103*, and *VaCML104* (Figure S5; Table S2) were analyzed together using one primer pair due to a high identity among the corresponding nucleotide sequences.

Water deficit was one of the strongest stimuli for induction of *VaCML* expression with a marked elevation in 1.8–13.1 times in transcript levels of nine genes (*VaCML62*, *VaCML72*, *VaCML79*, *VaCML83*, *VaCML92*, *VaCML93*, *VaCML105*, *VaCML106*, and *VaCML110*) detected after 12 h and 24 h of treatment (Figure 3, Figure 5, Figures S2 and S4). In addition, incubation under water deficit resulted in a progressive and considerable down-regulation in 1.7–3.1 times of three *CMLs*, including *VaCML57*, *VaCML66*, and *VaCML88*, which was detected after two periods of treatment (Figure 4, Figure 5, Figures S3 and S4).

Similar to the effect of water deficit, high salt stress considerably affected *VaCML* expression with both up- and down-regulation. Transcript levels of *VaCML44*, *VaCML61*, *VaCML78*, *VaCML82*, *VaCML86*, and *VaCML92* were significantly induced in 2.1–15 times at a minimum of two time intervals (Figure 3, Figure 5, Figures S2 and S4), while the transcript abundance of *VaCML57*, *VaCML60*, *VaCML77*, and *VaCML89* was progressively suppressed in 1.8–2.8 times at a minimum of two time intervals (Figure 4, Figure 5, Figures S3 and S4). Notably, the *VaCML44*, *VaCML61*, and *VaCML57* genes progressively and remarkably responded after 6 h, 12 h and 24 h of high salt exposure (Figure 3, Figure 4, Figures S2 and S3).

High mannitol stress had a weaker effect on *VaCML* gene expression in comparison with other abiotic stress treatments. We detected a considerable activation of only three *CMLs* (*VaCML79*, *VaCML92*, and *VaCML105*) in 1.9–5.9 times after two time periods of the high mannitol treatment (Figure 5 and Figure S4). As for negative regulation, only *VaCML57* gene was progressively down-regulated under high mannitol conditions (Figure 4 and Figure S3). Other *VaCML* genes responded with considerable changes only at one time interval post-treatment or remained unresponsive to the high mannitol treatment (Figure 3, Figure 4, Figure 5 and Figures S2–S4).

Heat and cold stress conditions markedly affected *VaCML* expression. In response to heat stress, expression of *VaCML1*, *VaCML21*, *VaCML22*, *VaCML52*, *VaCML107*, and *VaCML108* was distinctly modulated in 1.7–5.9 times at two time intervals post-treatment (Figure 3, Figure 5 and Figures S2 and S4), while expression of *VaCML60*, *VaCML79*, *VaCML88*, *VaCML89*, and *VaCML105* was progressively repressed under the high temperature conditions 6 h and 12 h after the treatment in most cases (Figure 4, Figure 5 and Figures S3 and S4). Incubation at lowered temperatures (+4 °C or +10 °C) strongly induced transcript levels of six *CML* genes (*VaCML21*, *VaCML44*, *VaCML61*, *VaCML78*, *VaCML86*, and *VaCML89*) and suppressed eight *CML* genes (*VaCML9a*, *VaCML48*, *VaCML57*, *VaCML75*, *VaCML82*, *VaCML85*, *VaCML92*, and *VaCML107*) at a minimum of two incubation intervals post-treatment (Figure 3, Figure 4 and Figure 5 and Figures S2–S4). Notably, although these low temperature stresses are similar, expression of only *VaCML61*, *VaCML75*, and *VaCML89* genes was significantly and progressively affected by both these incubation temperatures at least at two time periods of the cold treatments. Transcript levels of *VaCML44* and *VaCML92* markedly responded exclusively to the incubation at +10 °C, while transcript levels of *VaCML9a*, *VaCML57*, *VaCML85*, *VaCML86*, and *VaCML107* were affected only at +4 °C (Figure 3, Figure 4, Figure 5 and Figures S2–S4).

The data obtained revealed that the detected changes in transcription levels of 16 *CML* genes were not consistent and exhibited rather slight effects (*VaCML9b*, *VaCML41b*, *VaCML54*, *VaCML65*, *VaCML71*, *VaCML73*, *VaCML74*, *VaCML76*, and *VaCML90/91*), or considerable effects were observed only 24 h after stress application (*VaCML41a*, *VaCML81*, *VaCML95*, *VaCML96*, *VaCML100*, *VaCML103*, and *VaCML104*) (Figure S5). Expression of six *CML* genes (*VaCML51*, *VaCML53*, *VaCML80*, *VaCML87*, *VaCML94*, *VaCML109*) remained unresponsive to the abiotic stress treatments (Figure S6). We also noted that some genes originating from the same subgroup of the phylogenetic tree (Figure 1) showed similar expression pattern under the abiotic stress treatments. For example, *VaCML9a*, *VaCML79*, *VaCML107* (subgroup 2); *VaCML92*, *VaCML93*, *VaCML105*, *VaCML110* (subgroup 4); *VaCML83*, *VaCML84*, *VaCML89*, *VaCML106* (subgroup 6), *VaCML48*, and *VaCML82* (subgroup 7) (Figure 3, Figure 5, Figures S2 and S4).

## 3. Discussion

Wild grape *V. amurensis* is known to exhibit a high resistance to abiotic stresses, especially to cold stress [34]. Therefore, it would be interesting to investigate transcriptional responses of *V. amurensis* genes to various abiotic stimuli. After completion of sequencing and annotation of various plant genomes, the presence of *CaMs* and large *CML*-encoding gene families has emerged as a typical feature of plant genomes. The grapevine genome was not an exception. Recently, based on the analysis of the available grapevine genome sequencing data, Vandelle et al. [25] determined the presence of three *CaM* and 62 *CML* genes in the genome of the commonly cultivated grapevine *V. vinifera*. Using the publicly available gene expression datasets of different organs and tissues of *V. vinifera* cv. Corvina (clone 48), Vandelle et al. [25] analyzed expression of *VviCaMs* and *VviCMLs* in detail in the different grape organs and after application of drought, shade, heat, glucose, ultraviolet-C, and abscisic acid. In our study, we treated the stem cuttings of wild grape *V. amurensis* with a number of abiotic stress factors, including desiccation, high salinity, high mannitol, heat and cold stresses. The full-length coding sequences of *VaCaMs* and *VaCMLs* were cloned and their transcript levels were analyzed by real-time qRT-PCR after 6 h, 12 h, and 24 h of treatments.

We revised the identified *CaM* and *CML* genes in the *V. vinifera* genome [25] and described seven additional *VviCMLs* increasing the family size to 68 *VviCMLs* in *V. vinifera*. Then, we identified the *CaM* and *CML* genes expressed in wild grape *V. amurensis* in response to desiccation, high salinity, high mannitol, and temperature stresses. The analysis allowed identification and characterization of three *CaMs* and 54 *CMLs* of *V. amurensis* expressed in non-stressed tissues of *V. amurensis* and under the analyzed abiotic stress conditions. The qRT-PCR analysis of the *VaCaM* expression profiles revealed that the genes were actively expressed under the high salinity, high mannitol, desiccation stress, heat, and low temperature stress conditions. The applied abiotic stress treatments positively regulated expression of *CaM* genes in *V. amurensis*, with the most persistent changes being observed for *VaCaM8* in response to salt stress, but the alternations in *VaCaM* expression were not progressive and remarkable. The data suggest that *CaMs* are slightly implicated in the abiotic stress resistance of *V. amurensis*. In contrast to *VaCaMs*, most of the evaluated *VaCMLs* (32 *CMLs* out of 54 analyzed genes) were highly responsive to the analyzed abiotic stress conditions exhibiting both positive and negative regulation patterns. The results obtained indicated that the 32 *VaCMLs* play distinct positive and negative roles in responses of wild grape to abiotic stresses. The expression patterns of individual *VaCMLs* have been shown to be induced and/or repressed in response to particular stress stimuli and frequently varied temporally and in magnitude in response to the abiotic stresses, suggesting specificity in their roles in abiotic stress adaptation of *V. amurensis*. The expression patterns of some *VaCMLs* were similar under the analyzed stress conditions, which suggests that the genes could perform similar functions and/or are regulated by related molecular mechanisms. The other 22 *VaCMLs* displayed insensitive expression patterns, showed occasional regulation or were slightly affected, indicating that they are not implicated in *V. amurensis* abiotic stress responses.

The effects of heat stress on *CaM* and *CML* transcript levels were analyzed both *V. amurensis* in our study and for *V. vinifera* by Vandelle et al. [25]. The *CaM* and *CML* genes of *V. amurensis* and *V. vinifera* displayed similar regulation under heat application. For example, heat stress greatly increased expression of the *VviCML55* in *V. vinifera* and the homologous *VaCML55* gene in *V. amurensis*, while it negatively regulated *CML60*, *CML79*, and *CML92* expression in both these grapevine species. In addition, we noted that both *VaCML92* and *VvCML92* were strongly induced by water deficit stress [25].

Recently, a number of studies demonstrated that overexpression of plant *CaM* and *CML* genes can improve plant resistance to abiotic stresses [17,18,19,20]. For example, overexpression of the *ShCML44* gene isolated from cold tolerant wild tomato enhanced the tolerance of a stress-sensitive tomato to cold, drought, and salinity [18]. According to our data, a homologous *VaCML44* gene shared 66% protein similarity to *ShCML44* and was up-regulated under cold and salinity stress treatments (Figure 2). Overexpression of the *CsCaM3* gene, which shared 100% protein similarity with *VaCaM8* and *VaCaM10*, improved heat stress tolerance in cucumber [19]. Heat stress significantly increased transcript levels of the *VaCaM8* and *VaCaM10* genes after 6 h of treatment in our experiments (Figure 1).

Results obtained in the present study suggested that the *VaCML44*, *VaCML61*, *VaCML86*, and *VaCML89* genes were strongly and progressively induced under salt and cold stresses and are promising candidates for use in overexpression experiments to obtain plants with increased tolerance to these abiotic stress stimuli. The *VaCML21*, *VaCML22*, and *VaCML52* genes are in turn good candidates for increasing heat stress tolerance and *VaCML79*, *VaCML83*, *VaCML93*, 106, and *VaCML110*—for increasing water deficit tolerance. The assumptions need to be verified by establishing transgenic plants in future experiments.

In conclusion, our study revealed potential importance of a number of CaM/CML genes of *V. amurensis* in its adaptation to abiotic stresses and indicated that some *VaCaM* and *VaCML* genes (mainly presented in Figure 2 and Figure 4) are positive regulators of plant abiotic stress tolerance and can be used in plant biotechnology and molecular biology in overexpression experiments to obtain plants with increased resistance to water-deficit, salinity, osmoticum, and temperature stresses. The *VaCML* genes with negative responses to the abiotic stimuli may be blockers in the development of the wild grape stress signaling and need additional studies.

## 4. Materials and Methods

### 4.1. Plant Material and Treatments

For the abiotic stress treatments, we used young vines of wild-growing grapevine *V. amurensis* Rupr. (*Vitaceae*) sampled from a non-protected natural population near Vladivostok, Russia (Akademgorodok, the southern Primorsky region of the Russian Far East, longitude 43.2242327 and latitude 131.99112300). The vines were collected in August 2018 and identified at the Botany Department of the Federal Scientific Center of the East Asia Terrestrial Biodiversity. The *V. amurensis* vines were divided into cuttings (excised young stems 7–8 cm long with one healthy leaf) that were placed in individual beakers and used for the stress treatments. For the control non-stress treatment, the *V. amurensis* cuttings were placed in filtered water at 25 °C. To induce water deficit stress, the cuttings were laid on a paper towel at 25 °C. To induce osmotic stress, the cuttings were placed in 400 mМ NaCl and 400 mМ mannitol solutions at 25 °C. To apply cold and heat stress, the *V. amurensis* cuttings were placed in filtered water in the growth chamber (Sanyo MLR-352, Panasonic, Osaka, Japan) at +4 °C, +10 °C, and +37 °C. The *V. amurensis* cuttings were grown under a 16/8 h light/dark phoperiod. The freshly harvested cuttings were acclimated to the “non-stress” condition for 30 min before they were treated with the stress treatments. The experiments were repeated three times for each stress treatment time and for the control treatment.

### 4.2. RNA Isolation and cDNA Preparation

Leaf samples were harvested after 6 h, 12 h, and 24 h of the abiotic stress treatments and immediately used for RNA extraction. Total RNA extraction was performed using the cetyltrimethylammonium bromide (CTAB)-based extraction as described [35]. Complementary DNAs were synthesized using 1.5 µg of RNA by the Moloney Murine Leukemia Virus (MMLV) Reverse transcription PCR Kit (RT-PCR, Sileks M, Moscow, Russia) as described [36]. The reverse transcription products were amplified by PCR and verified on the absence for DNA contamination using primers for *AtActin2* gene (NM_112764) listed in Table S1 (356 bp PCR product from cDNA and 442 bp from DNA).

### 4.3. Cloning and Sequencing of VaCaM and VaCML Transcripts

To clone and sequence cDNAs containing full-length CDS of the *VaCaM* and *VaCML* transcripts, we retrieved the predicted protein and mRNA sequences of *V. vinifera* CaMs and CMLs from the Grape Genome Database hosted at CRIBI [37]. For this purpose, the CaM and CML protein sequences of *Arabidopsis thaliana* were downloaded from the TAIR database [38] and used as queries for blastp (V2 gene prediction) search against the deduced proteome of the PN40024 *V. vinifera* genome as described [25]. For non-redundant protein sequences, we performed a domain analysis by PROSITE scan [29,39] and prediction of myristoylation and palmitoylation motif numbers with GPS-Lipid [31,40]. The coding nucleotide sequences of the selected *V. vinifera CaMs* and *CMLs* were downloaded from the CRIBI database using tblastn search with the VviCaMs and VviCMLs protein sequences as queries (V2 mRNA prediction). Specific primers for amplification of the full-length coding cDNA sequences of the *VaCaM* and *VaCML* transcripts (Table S1) were designed to the retrieved VviCaMs and VviCMLs.

The coding cDNA sequences of *VaCaMs* and *VaCMLs* were amplified using RNA extracted from unstressed leaves of *V. amurensis*. To clone the full-length cDNAs of the *VaCaMs* and *VaCMLs*, RT-PCRs were performed in a T100^TM^ Thermal Cycler (Bio-Rad Laboratories, Inc., Hercules, CA, USA) in 20 µL aliquots of the reaction mixtures using Ta 50–56 °C, elongation time 40 s—1 min 30 s. For the PCR reactions, we used Pfu polymerase (Sileks M, Moscow, Russia) as described in Kiselev et al. [41].

The obtained PCR products of *VaCaM* and *VaCML* cDNAs were subcloned into a pJET1.2 using CloneJET PCR Cloninig Kit (ThermoFisher Scientific, Waltham, MA, USA) and sequenced using an ABI 3130 Genetic Analyzer (Applied Biosystems, Foster City, CA, USA) according to the manuphacturer’s instructions. The sequences of the *V. amurensis VaCaM* and *VaCML* transcripts were deposited to GenBank (Table 1).

Multiple sequence alignments were done with the ClustalX program [42]. For classification of the CaMs and CMLs into subfamilies, a phylogenetic tree was created with the Clustal Omega program [43]. The VaCaM and VaCML amino acid sequences were predicted using the Gene Runner 3.05 program. The following online websites were used to predict molecular weights, N-terminal myristoylation and palmitoylation motifs, and domain structure of the VaCaM and VaCML proteins: Compute pI/Mw tool [32], GPS-Lipid [31,40], and PROSITE [29].

### 4.4. Expression Analysis of VaCaMs and VaCMLs

Quantitative RT-PCR (qRT-PCR) was performed using Real-time PCR kit (Syntol, Moscow, Russia) and EvaGreen Real-time PCR dye (Biotium, Hayward, CA, USA) using cDNAs of *VaCaMs* and *VaCMLs* and two internal controls (*VaGAPDH* and *VaActin1*) as described [44,45]. The expression was calculated by the 2^−ΔΔCT^ method [46]. The visualized heatmaps were generated using heatmapper.ca [47,48]. Primers used for qRT-PCRsare listed in Table S2. qRT-PCR data shown were obtained from three independent experiments.

### 4.5. Statistical Analysis

The statistical analysis was carried out using the Microsoft Office Excel 2007 program (Microsoft corporation, Redmond, WA, USA). The data are presented as mean ± standard error (SE) and were tested by paired Student’s *t*-test. The 0.05 level was selected as the point of minimal statistical significance in all analyses.

## Figures and Tables

**Table 1:** Characteristics of *CaM* and *CML* transcripts and deduced amino acid sequences of *Vitis amurensis* and *Vitis vinifera*.

*Vitis amurensis* cDNA Clone				*Vitis vinifera* Gene Prediction				Protein I/S (%)	No. of EF Hands	N-Myrist	N-Palmit	MW (kDa)	Group
Transcript Name	ID GeneBank	CDS (bp)	Amino Acids	Gene Name	Gene ID	CDS (bp)	Amino Acids						
*VaCaM8*	MN515154	450	149	*VviCaM8*	VIT_208s0040g00470.6	450	149	100/100	4	-	-	16.8	1
*VaCaM9*	MN478368	450	149	*VviCaM9*	VIT_217s0000g00580.1	450	149	100/100	4	-	-	16.9	1
*VaCaM10*	MN515156	450	149	*VviCaM10*	VIT_206s0009g01910.1	462	153	100/100	4	-	-	16.8	1
*VaCML9a*	MN515159	462	153	*VviCML9a*	VIT_214s0030g02150.1	462	153	97/98	4	-	-	17.5	2
*VaCML9b*	MN515160	450	149	*VviCML9b*	VIT_200s0179g00280.1	450	149	100/100	4	-	Yes	17.0	2
*VaCML79*	MN515161	450	149	*VviCML79*	VIT_205s0020g04420.1	450	149	100/100	4	-	-	17.0	2
*VaCML80*	MN515162	444	147	*VviCML80*	VIT_202s0241g00140.1	444	147	100/100	3	Yes	-	16.6	2
*VaCML107*	MN562253	438	145	*VviCML107*	VIT_207s0031g00700.3	438	145	99/98	3	-	Yes	16.8	2
*VaCML108*	MN562252	522	173	*VviCML108*	VIT_205s0094g01240.3	522	173	99/99	4	-	-	19.9	2
*VaCML72*	MN515163	483	160	*VviCML72*	VIT_217s0000g04460.1	483	160	99/99	4	Yes	-	17.6	3
*VaCML73*	MN515164	489	162	*VviCML73*	VIT_201s0011g02470.1	489	162	100/100	4	-	-	17.8	3
*VaCML74*	MN537892	492	163	*VviCML74*	VIT_216s0039g01880.1	492	163	100/100	4	-	-	18.2	3
*VaCML75*	MN537893	492	163	*VviCML75*	VIT_202s0012g02060.1	492	163	100/100	4	-	-	18.1	3
*VaCML1*	MN537894	552	183	*VviCML1*	VIT_203s0063g00530.1	549	182	98/98	4	-	-	21.0	4
*VaCML41a*	MN537895	561	186	*VviCML41a*	VIT_204s0023g01100.1	558	185	97/98	3	Yes	-	21.0	4
*VaCML41b*	MN537896	576	191	*VviCML41b*	VIT_218s0001g11830.1	576	191	98/98	3	-	-	21.2	4
*VaCML44*	MN537897	489	162	*VviCML44*	VIT_218s0001g01630.1	489	162	99/99	4	-	-	18.3	4
*VaCML60*	MN537898	669	222	*VviCML60*	VIT_208s0007g05790.1	669	222	98/99	4	-	-	24.3	4
*VaCML86*	MN540577	492	163	*VviCML86*	VIT_217s0000g02480.1	282	93	98/98 ^a^	4	-	-	18.1	4
*VaCML88*	MN540578	579	192	*VviCML88*	VIT_208s0056g00290.1	579	192	100/100	3	Yes	-	21.4	4
*VaCML89*	MN540579	663	220	*VviCML89*	VIT_211s0016g05740.1	771	256	99/100	4	-	Yes	24.5	4
*VaCML90*	MN540580	465	154	*VviCML90*	VIT_207s0289g00040.1	465	154	100/100	4	-	Yes	17.5	4
*VaCML91*				*VviCML91*	VIT_207s0141g00300.1								
*VaCML92*	MN540581	507	168	*VviCML92*	VIT_218s0122g00180.1	507	168	100/100	4	-	Yes	18.4	4
*VaCML93*	MN540582	429	142	*VviCML93*	VIT_214s0171g00150.1	429	142	99/99	4	-	-	15.9	4
*VaCML94*	MN540583	432	143	*VviCML94*	VIT_217s0000g01630.1	432	143	99/100	4	-	-	16.1	4
*VaCML105*	MN562248	453	150	*VviCML105*	VIT_214s0006g01400.1	453	150	100/100	4	-	Yes	16.5	4
*VaCML106*	MN562254	255	84	*VviCML106*	VIT_208s0007g08830.1	255	84	100/100	2	-	-	9.5	4
*VaCML109*	MN562249	615	204	*VviCML109*	VIT_215s0048g00790.1	615	204	99/99	4	-	Yes	22.9	4
*VaCML110*	MN562246	648	215	*VviCML110*	VIT_205s0102g00450.1	645	214	99/99	4	-	Yes	24.6	4
*VaCML95*	MN540584	423	140	*VviCML95*	VIT_201s0010g03000.1	423	140	98/99	4	-	Yes	16.1	5
*VaCML96*	MN540585	423	140	*VviCML96*	VIT_201s0010g02990.1	423	140	96/97	4	-	Yes	16.1	5
nd				*VviCML97*	VIT_201s0010g02940.1	423				-	-	16.1	5
nd				*VviCML98*	VIT_201s0010g02970.1	423				-	-	16.1	5
nd				*VviCML99*	VIT_201s0010g03010.1	423				-	-	16.1	5
*VaCML100*	MN540586	423	140	*VviCML100*	VIT_201s0010g02980.1	423	140	96/97	4	-	Yes	16.1	5
nd				*VviCML101*	VIT_201s0010g02930.1	423				-	-	16.1	5
nd				*VviCML102*	VIT_201s0010g02950.1	423				-	-	16.1	5
*VaCML103*	MN540587	423	140	*VviCML103*	VIT_201s0010g03040.1	423	140	95/96	4	-	Yes	16.1	5
*VaCML104*	MN540588	423	140	*VviCML104*	VIT_201s0010g03020.1	423	140	96/97	4	-	Yes	16.1	5
*VaCML55*	MN540589	294	97	*VviCML55*	VIT_218s0001g10670.1	294	97	100/100	2	-	Yes	11.4	6 ^a^
*VaCML83*	MN540590	543	180	*VviCML83*	VIT_201s0026g02590.1	543	180	98/98	2	-	Yes	20.5	6 ^a^
*VaCML84*	MN540591	471	156	*VviCML84*	VIT_214s0108g01000.1	471	156	99/99	2	-	Yes	18.3	6 ^a^
*VaCML85*	MN540592	591	196	*VviCML85*	VIT_217s0000g06325.1	591	196	99/99	2	-	Yes	22.0	6 ^a^
*VaCML87*	MN540593	294	97	*VviCML87*	VIT_207s0031g00760.1	294	97	99/98	2	-	Yes	11.5	6 ^a^
*VaCML51*	MN540594	288	95	*VviCML51*	VIT_218s0001g10605.1	288	95	96/98	2	-	-	10.6	6 ^b^
*VaCML52*	MN540595	279	92	*VviCML52*	VIT_218s0001g10600.1	279	92	100/100	2	-	-	10.6	6 ^b^
*VaCML53*	MN540596	270	89	*VviCML53*	VIT_218s0001g10595.1	270	89	98/100	2	-	-	10.6	6 ^b^
*VaCML54*	MN540597	270	89	*VviCML54*	VIT_218s0001g10645.1	270	89	98/98	2	-	Yes	10.3	6 ^b^
nd				*VviCML56*	VIT_218s0001g10630.1								6 ^b^
*VaCML57*	MN540598	294	97	*VviCML57*	VIT_218s0001g10620.1	285	94	97/96	2	-	-	11.2	6 ^b^
nd				*VviCML58*	VIT_218s0001g10640.1	435							6 ^b^
nd				*VviCML59*	VIT_218s0001g10610.1	300							6 ^b^
nd				*VviCML21v.1*	VIT_219s0015g01200.1	708							
*VaCML21v.2*	MN540599	699	232	*VviCML21v.2*	VIT_219s0015g01200.2	699	232	99/99	4	Yes	Yes	26.4	7 ^a^
*VaCML22*	MN540602	729	242	*VviCML22*	VIT_205s0029g00070.1	747	248	97/97	4	Yes	Yes	27.3	7 ^a^
*VaCML62*	MN540605	414		*VviCML62*	VIT_212s0059g00360.1	414		97/98	2	Yes	-	15.5	7 ^a^
nd				*VviCML63*	VIT_212s0059g00320.1	414							7 ^a^
nd				*VviCML64*	VIT_212s0059g00370.1	414							7 ^a^
*VaCML65*	MN540606	414	137	*VviCML65*	VIT_212s0059g00430.1	414	137	99/99	2	Yes	-	15.6	7 ^a^
*VaCML66*	MN540607	420	139	*VviCML66*	VIT_213s0156g00120.1	420	139	100/100	2	Yes	-	15.5	7 ^a^
nd				*VviCML67*	VIT_212s0059g00340.1	414							7 ^a^
nd				*VviCML68*	VIT_212s0059g00420.1	414							7 ^a^
nd				*VviCML69*	VIT_212s0059g00400.1	414							7 ^a^
nd				*VviCML70*	VIT_212s0059g00350.1	414							7 ^a^
*VaCML48*	MN562247	678	225	*VviCML48*	VIT_206s0080g00450.1	678	225	100/100	2	-	-	25.3	7 ^b^
*VaCML61*	p.s.^b^			*VviCML61*	VIT_205s0077g00300.1	909	302	100/100	2	Yes	-	33.5	7 ^b^
nd				*VviCML77v.1*	VIT_200s0252g00130.1	711							7 ^b^
*VaCML77v.2*	MN540608	831	276	*VviCML77v.2*	VIT_200s0252g00130.2	831	276	98/98	2	Yes	-	29.7	
*VaCML78*	MN540610	1119	372	*VviCML78*	VIT_210s0071g00670.1	1119	372	98/99	5	Yes	-	43.4	7 ^b^
*VaCML81*	MN540611	1425	474	*VviCML81*	VIT_204s0008g06280.1	1425	474	99/99	4	-	Yes	54.2	7 ^b^
*VaCML82*	MN540612	1065	354	*VviCML82*	VIT_211s0118g00540.1	1065	354	99/100	5	-	-	40.6	7 ^b^
*VaCML71*	MN548771	594	72	*VviCML71*	VIT_219s0014g04650.1	453	150	100/100 ^a^	0	-	-	8.0	8
*VaCML76*	MN540595	363	120	*VviCML76*	VIT_202s0012g00660.1	363	120	99/99	1	Yes	-	13.5	8

Note: No. of EF hands—the number of EF hands predicted by PROSITE scan [28,29]; N-Myrist and N-Palmit—the number of myristoylation and palmitoylation motifs identified with GPS-Lipid [30,31]; CDS—coding DNA sequences; MW (kDa)—molecular mass calculated using the Compute pI/Mw tool [32]; I and S—identities and similarities of the deduced *V. amurensis* and *V. vinifera* amino acid sequences. ^a^ the protein I and S were obtained without taking into account insertions; ^b^ partially sequenced (Figure S7).

## Supplementary Materials

The following are available online at https://www.mdpi.com/2223-7747/8/12/602/s1. Table S1: Primers used for amplification of full-length cDNA of calmodulin (*CaM*), calmodulin-like (*CML*) genes, and partial cDNA of house-keeping gene (*AtActin2*) in wild-growing grapevine *Vitis amurensis*; Table S2: Primers used for real-time PCR for calmodulin (*CaM*) and calmodulin-like (*CML*) genes in wild-growing grapevine *Vitis amurensis*; Figure S1. Expression of *VaCaM8* (a), *VaCaM9* (b), and *VaCaM10* (c) genes 6 h, 12 h, and 24 h post-treatment in *V. amurensis* cuttings exposed to abiotic stress conditions. The *VaCaM* expression levels were determined by qRT-PCR. Control—non-stress conditions (filtered water, +25 °C); WD—water-deficit stress (cuttings laid on a paper towel, +25 °C); NaCl—salt stress (0.4 M NaCl, +25 °C); Mannitol—osmoticum (0.4 M mannitol, +25 °C); +37 °C—heat stress (filtered water, +37 °C); +10 °C and +4 °C—cold stress (filtered water, +10 °C and +4 °C). *, **—significantly different from the values of *CaM* expression in *V. amurensis* under the control conditions after 6 h, 12 h, or 24 h of treatments at *P* ≤ 0.05 and 0.01 according to the Student’s t-test; Figure S2. Expression of *VaCML21* (a), *VaCML22* (b), *VaCML44* (c), *VaCML52* (d), *VaCML61* (e), *VaCML62* (f), *VaCML72* (g), *VaCML83* (h), *VaCML86* (i), *VaCML93* (j), *VaCML106* (k), *VaCML108* (l), and *VaCML110* (m) genes 6 h, 12 h, and 24 h post-treatment in *V. amurensis* cuttings exposed to abiotic stress conditions. The *VaCaM* expression levels were determined by qRT-PCR. Control—non-stress conditions (filtered water, +25 °C); WD—water-deficit stress (cuttings laid on a paper towel, +25 °C); NaCl—salt stress (0.4 M NaCl, +25 °C); Mannitol—osmoticum (0.4 M mannitol, +25 °C); +37 °C—heat stress (filtered water, +37 °C); +10 °C and +4 °C—cold stress (filtered water, +10 °C and +4 °C). *, **—significantly different from the values of *CaM* expression in *V. amurensis* under the control conditions after 6 h, 12 h, or 24 h of treatments at *P* ≤ 0.05 and 0.01 according to the Student’s t-test; Figure S3. Expression of *VaCML57* (a), *VaCML60* (b), *VaCML66* (c), *VaCML77* (d), and *VaCML85* (e) genes 6 h, 12 h, and 24 h post-treatment in *V. amurensis* cuttings exposed to abiotic stress conditions. The *VaCaM* expression levels were determined by qRT-PCR. Control—non-stress conditions (filtered water, +25 °C); WD—water-deficit stress (cuttings laid on a paper towel, +25 °C); NaCl—salt stress (0.4 M NaCl, +25 °C); Mannitol—osmoticum (0.4 M mannitol, +25 °C); +37 °C—heat stress (filtered water, +37 °C); +10 °C and +4 °C—cold stress (filtered water, +10 °C and +4 °C). *, **—significantly different from the values of *CaM* expression in *V. amurensis* under the control conditions after 6 h, 12 h, or 24 h of treatments at *P* ≤ 0.05 and 0.01 according to the Student’s t-test; Figure S4. Expression of *VaCML1* (a), *VaCML9a* (b), *VaCML48* (c), *VaCML55* (d), *VaCML75* (e), *VaCML78* (f), *VaCML79* (g), *VaCML82* (h), *VaCML84* (i), *VaCML88* (j), *VaCML89* (k), *VaCML92* (l), *VaCML105* (m), and *VaCML107* (n) genes 6 h, 12 h, and 24 h post-treatment in *V. amurensis* cuttings exposed to abiotic stress conditions. The *VaCaM* expression levels were determined by qRT-PCR. Control—non-stress conditions (filtered water, +25 °C); WD—water-deficit stress (cuttings laid on a paper towel, +25 °C); NaCl—salt stress (0.4 M NaCl, +25 °C); Mannitol—osmoticum (0.4 M mannitol, +25 °C); +37 °C—heat stress (filtered water, +37 °C); +10 °C and +4 °C—cold stress (filtered water, +10 °C and +4 °C). *, **—significantly different from the values of *CaM* expression in *V. amurensis* under the control conditions after 6 h, 12 h, or 24 h of treatments at *P* ≤ 0.05 and 0.01 according to the Student’s t-test; Figure S5. Expression of *VaCML9b* (a), *VaCML41a* (b), *VaCML41b* (c), *VaCML54* (d), *VaCML65* (e), *VaCML71* (f), *VaCML73* (g), *VaCML74* (h), *VaCML76* (i), *VaCML81* (j), *VaCML95, 95, 100, 103, 104* (k), and *VaCML90, 91* (h) genes 6 h, 12 h, and 24 h post-treatment in *V. amurensis* cuttings exposed to abiotic stress conditions. The *VaCaM* expression levels were determined by qRT-PCR. Control—non-stress conditions (filtered water, +25 °C); WD—water-deficit stress (cuttings laid on a paper towel, +25 °C); NaCl—salt stress (0.4 M NaCl, +25 °C); Mannitol—osmoticum (0.4 M mannitol, +25 °C); +37 °C—heat stress (filtered water, +37 °C); +10 °C and +4 °C—cold stress (filtered water, +10 °C and +4 °C). *, **—significantly different from the values of *CaM* expression in *V. amurensis* under the control conditions after 6 h, 12 h, or 24 h of treatments at *P* ≤ 0.05 and 0.01 according to the Student’s t-test; Figure S6. Expression of *VaCML51* (a), *VaCML53* (b), *VaCML80* (c), *VaCML87* (d), *VaCML94* (e), and *VaCML109* (f) genes 6 h, 12 h, and 24 h post-treatment in *V. amurensis* cuttings exposed to abiotic stress conditions. The *VaCaM* expression levels were determined by qRT-PCR. Control—non-stress conditions (filtered water, +25 °C); WD—water-deficit stress (cuttings laid on a paper towel, +25 °C); NaCl—salt stress (0.4 M NaCl, +25 °C); Mannitol—osmoticum (0.4 M mannitol, +25 °C); +37 °C—heat stress (filtered water, +37 °C); +10 °C and +4 °C—cold stress (filtered water, +10 °C and +4 °C). *, **—significantly different from the values of *CaM* expression in *V. amurensis* under the control conditions after 6 h, 12 h, or 24 h of treatments at *P* ≤ 0.05 and 0.01 according to the Student’s t-test; Figure S7. DNA sequence alignment and partial nucleotide sequences of the *VviCML61* (VIT_205s0077g00300.1) and partially sequenced *VaCML61*.
