# Supplementary material for: The Effect of Abiotic Stress Conditions on Expression of Calmodulin (CaM) and Calmodulin-Like (CML) Genes in Wild-Growing Grapevine Vitis amurensis
Source: Plants (Basel). 2019 Dec 13;8(12):602. doi: 10.3390/plants8120602 (PMC6963546; doi:10.3390/plants8120602)
Supplement: Supplementary file 1 [file plants-08-00602-s001.zip › plants-664661-supplementary/plants-664661-supplementary/Supplementary-664661/Supplementary Figures.docx]

**Figure S1.** Expression of *VaCaM8* (a), *VaCaM9* (b), and *VaCaM10* (c) genes 6 h, 12 h, and 24 h post-treatment in *V. amurensis* cuttings exposed to abiotic stress conditions. The *VaCaM* expression levels were determined by qRT-PCR. Control – non-stress conditions (filtered water, +25^o^C); WD – water-deficit stress (cuttings laid on a paper towel, +25^o^C); NaCl – salt stress (0.4 M NaCl, +25^o^C); Mannitol – osmoticum (0.4 M mannitol, +25oC); +37^o^C – heat stress (filtered water, +37^o^C); +10oC and +4^o^C – cold stress (filtered water, +10^o^C and +4^o^C). *, **—signiﬁcantly different from the values of *CaM* expression in *V. amurensis* under the control conditions after 6 h, 12 h, or 24 h of treatments at *P* ≤ 0.05 and 0.01 according to the Student’s t-test.

**
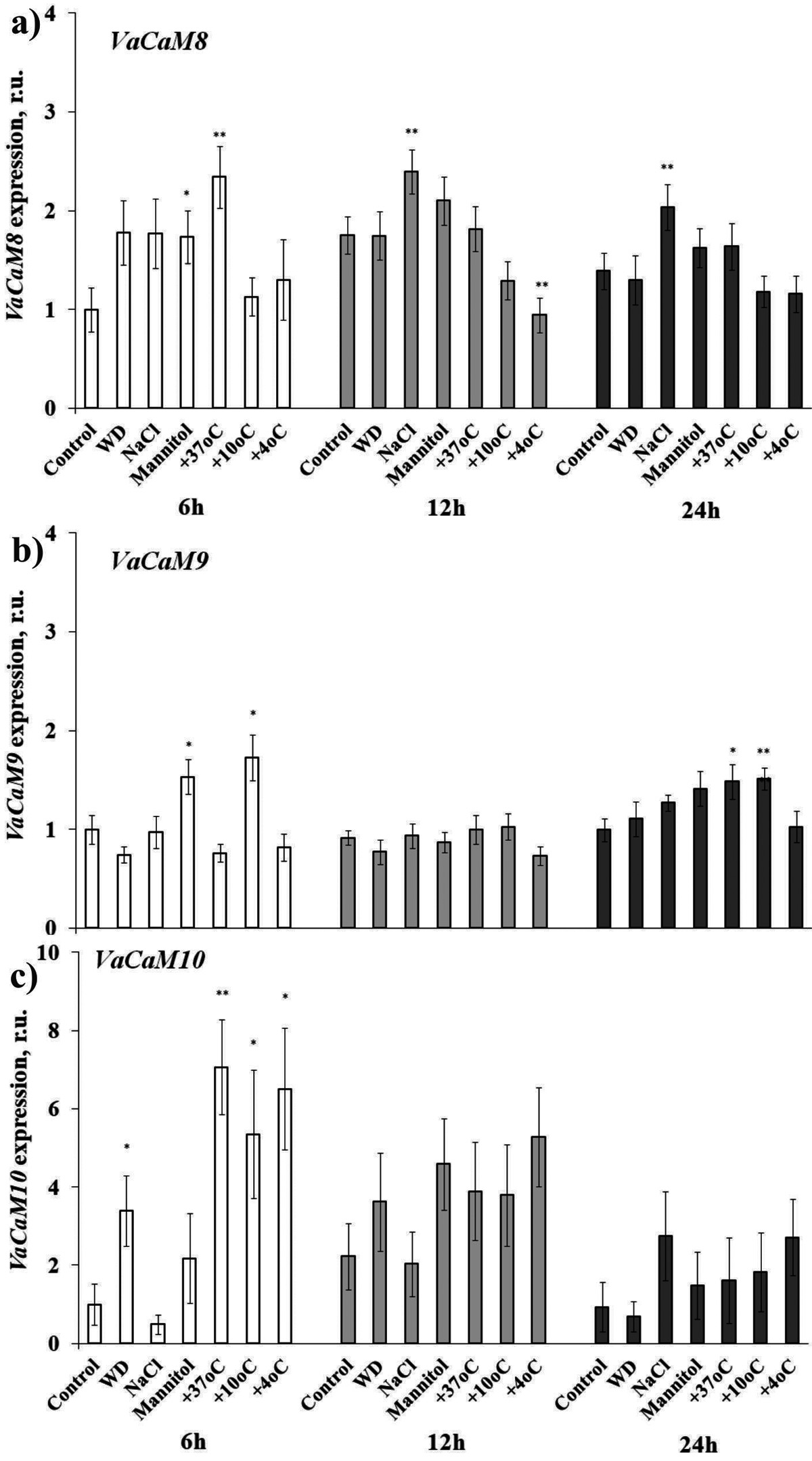
**

**Figure S2.** Expression of *VaCML21* (a), *VaCML22* (b), *VaCML44* (c), *VaCML52* (d), *VaCML61* (e), *VaCML62* (f), *VaCML72* (g), *VaCML83* (h), *VaCML86* (i), *VaCML93* (j), *VaCML106* (k), *VaCML108* (l), and *VaCML110* (m) genes 6 h, 12 h, and 24 h post-treatment in *V. amurensis* cuttings exposed to abiotic stress conditions. The *VaCaM* expression levels were determined by qRT-PCR. Control – non-stress conditions (filtered water, +25^o^C); WD – water-deficit stress (cuttings laid on a paper towel, +25^o^C); NaCl – salt stress (0.4 M NaCl, +25^o^C); Mannitol – osmoticum (0.4 M mannitol, +25^o^C); +37^o^C – heat stress (filtered water, +37^o^C); +10^o^C and +4^o^C – cold stress (filtered water, +10^o^C and +4^o^C). *, **—signiﬁcantly different from the values of *CaM* expression in *V. amurensis* under the control conditions after 6 h, 12 h, or 24 h of treatments at *P* ≤ 0.05 and 0.01 according to the Student’s t-test.

**
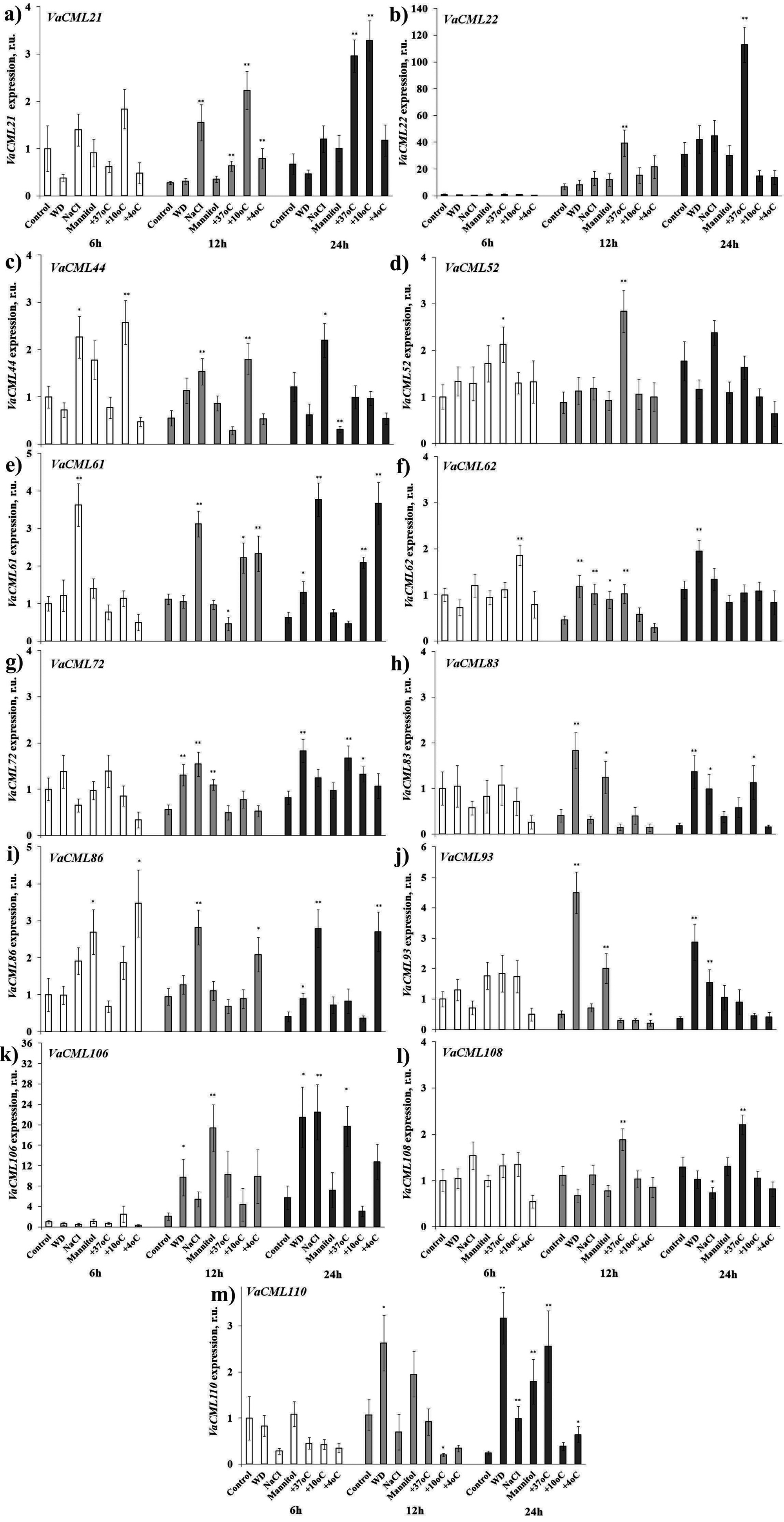
**

**Figure S3.** Expression of *VaCML57* (a), *VaCML60* (b), *VaCML66* (c), *VaCML77* (d), and *VaCML85* (e) genes 6 h, 12 h, and 24 h post-treatment in *V. amurensis* cuttings exposed to abiotic stress conditions. The *VaCaM* expression levels were determined by qRT-PCR. Control – non-stress conditions (filtered water, +25^o^C); WD – water-deficit stress (cuttings laid on a paper towel, +25^o^C); NaCl – salt stress (0.4 M NaCl, +25^o^C); Mannitol – osmoticum (0.4 M mannitol, +25^o^C); +37^o^C – heat stress (filtered water, +37^o^C); +10^o^C and +4^o^C – cold stress (filtered water, +10^o^C and +4^o^C). *, **—signiﬁcantly different from the values of *CaM* expression in *V. amurensis* under the control conditions after 6 h, 12 h, or 24 h of treatments at *P* ≤ 0.05 and 0.01 according to the Student’s t-test.

**
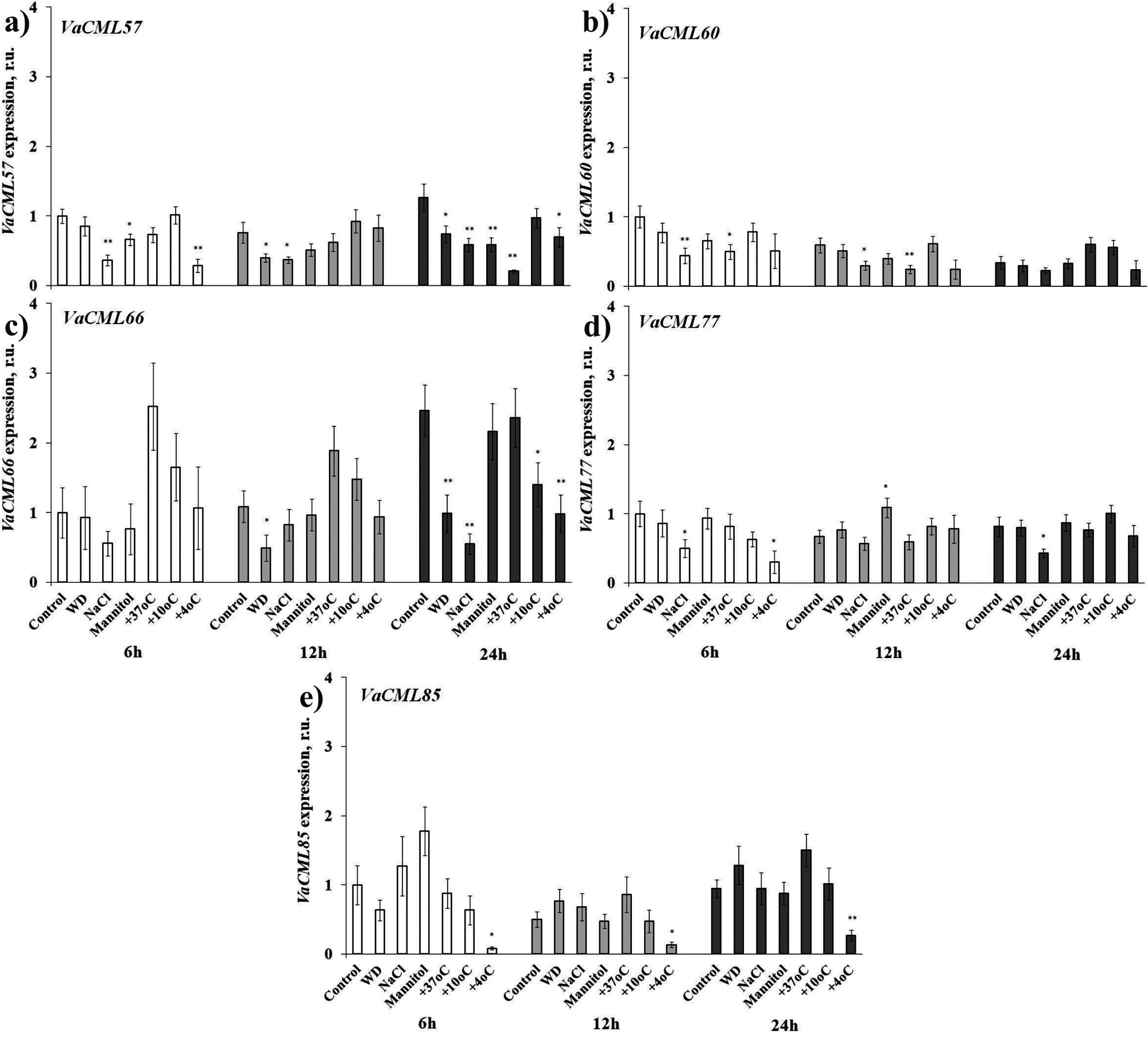
**

**Figure S4.** Expression of *VaCML1* (a), *VaCML9a* (b), *VaCML48* (c), *VaCML55* (d), *VaCML75* (e), *VaCML78* (f), *VaCML79* (g), *VaCML82* (h), *VaCML84* (i), *VaCML88* (j), *VaCML89* (k), *VaCML92* (l), *VaCML105* (m), and *VaCML107* (n) genes 6 h, 12 h, and 24 h post-treatment in *V. amurensis* cuttings exposed to abiotic stress conditions. The *VaCaM* expression levels were determined by qRT-PCR. Control – non-stress conditions (filtered water, +25^o^C); WD – water-deficit stress (cuttings laid on a paper towel, +25^o^C); NaCl – salt stress (0.4 M NaCl, +25^o^C); Mannitol – osmoticum (0.4 M mannitol, +25^o^C); +37^o^C – heat stress (filtered water, +37^o^C); +10^o^C and +4^o^C – cold stress (filtered water, +10^o^C and +4^o^C). *, **—signiﬁcantly different from the values of *CaM* expression in *V. amurensis* under the control conditions after 6 h, 12 h, or 24 h of treatments at *P* ≤ 0.05 and 0.01 according to the Student’s t-test.


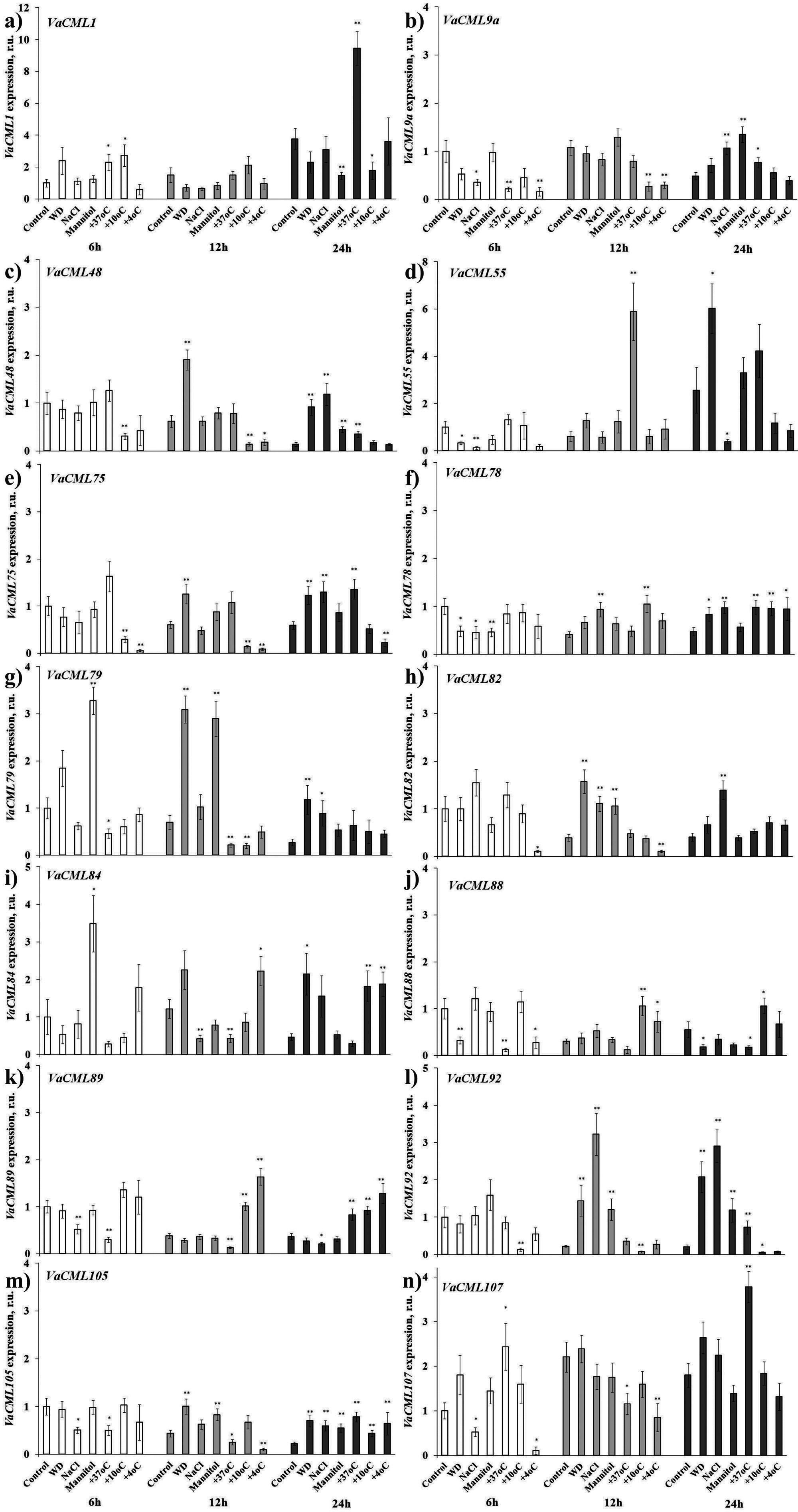


**Figure S5.** Expression of *VaCML9b* (a), *VaCML41a* (b), *VaCML41b* (c), *VaCML54* (d), *VaCML65* (e), *VaCML71* (f), *VaCML73* (g), *VaCML74* (h), *VaCML76* (i), *VaCML81* (j), *VaCML95, 95, 100, 103, 104* (k), and *VaCML90, 91* (h) genes 6 h, 12 h, and 24 h post-treatment in *V. amurensis* cuttings exposed to abiotic stress conditions. The *VaCaM* expression levels were determined by qRT-PCR. Control – non-stress conditions (filtered water, +25^o^C); WD – water-deficit stress (cuttings laid on a paper towel, +25^o^C); NaCl – salt stress (0.4 M NaCl, +25^o^C); Mannitol – osmoticum (0.4 M mannitol, +25^o^C); +37^o^C – heat stress (filtered water, +37^o^C); +10^o^C and +4^o^C – cold stress (filtered water, +10^o^C and +4^o^C). *, **—signiﬁcantly different from the values of *CaM* expression in *V. amurensis* under the control conditions after 6 h, 12 h, or 24 h of treatments at *P* ≤ 0.05 and 0.01 according to the Student’s t-test.

**
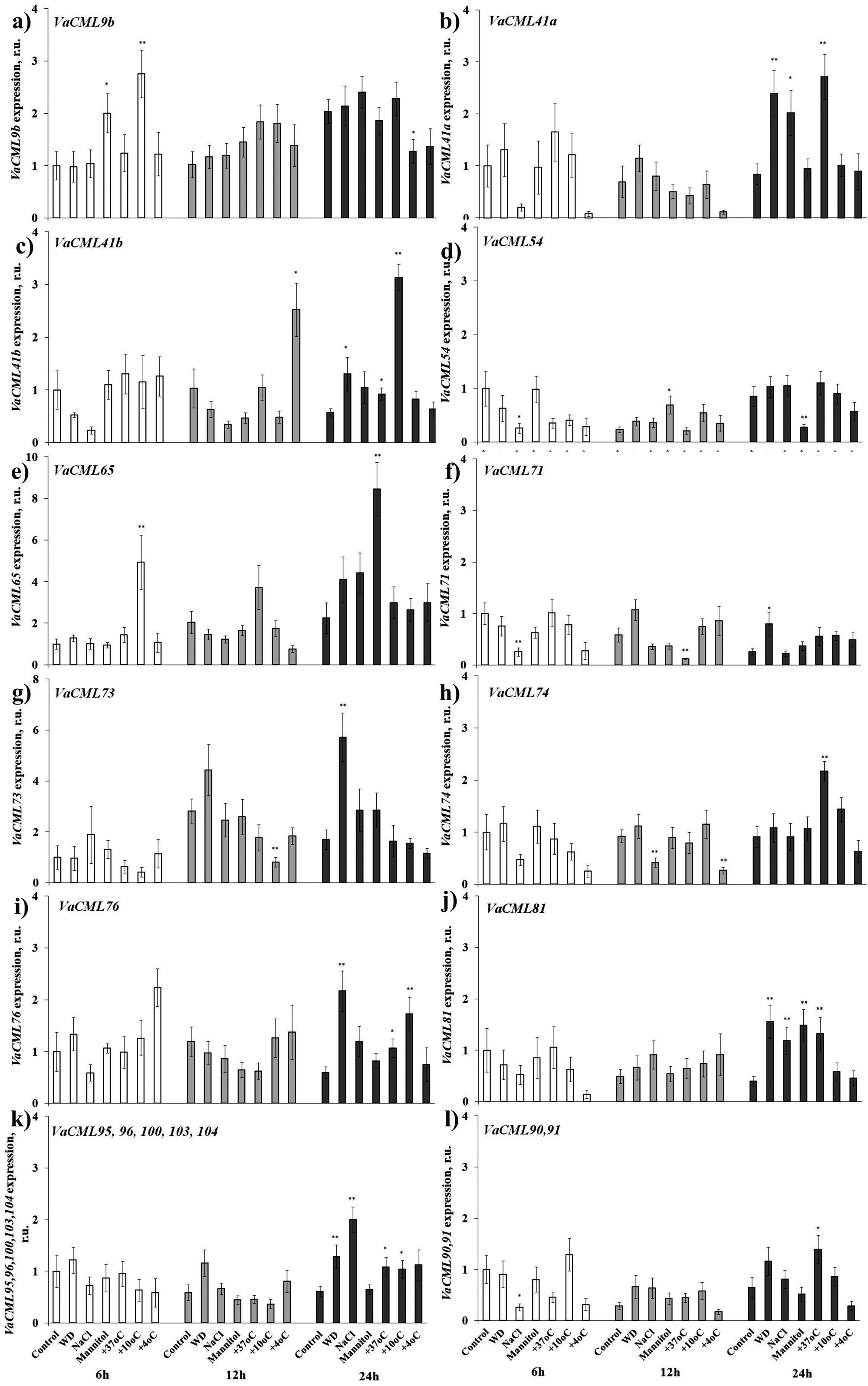
**

**Figure S6.** Expression of *VaCML51* (a), *VaCML53 (*b), *VaCML80* (c), *VaCML87* (d), *VaCML94* (e), and *VaCML109* (f) genes 6 h, 12 h, and 24 h post-treatment in *V. amurensis* cuttings exposed to abiotic stress conditions. The *VaCaM* expression levels were determined by qRT-PCR. Control – non-stress conditions (filtered water, +25^o^C); WD – water-deficit stress (cuttings laid on a paper towel, +25^o^C); NaCl – salt stress (0.4 M NaCl, +25^o^C); Mannitol – osmoticum (0.4 M mannitol, +25^o^C); +37^o^C – heat stress (filtered water, +37^o^C); +10^o^C and +4^o^C – cold stress (filtered water, +10^o^C and +4^o^C). *, **—signiﬁcantly different from the values of *CaM* expression in *V. amurensis* under the control conditions after 6 h, 12 h, or 24 h of treatments at *P* ≤ 0.05 and 0.01 according to the Student’s t-test.


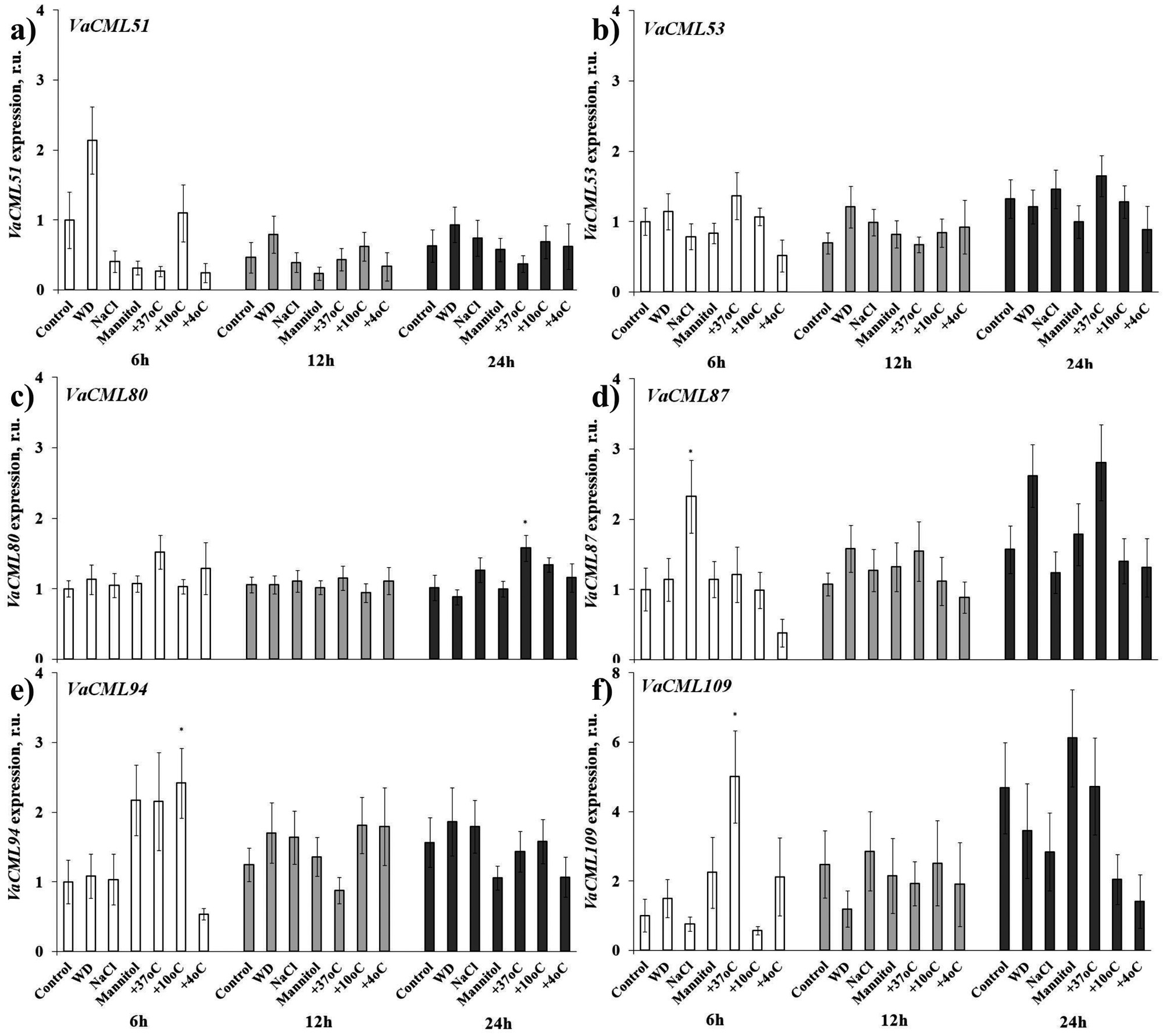


**Figure S7.** DNA sequence alignment and partial nucleotide sequences of the *VviCML61* (VIT_205s0077g00300.1) and partially sequenced *VaCML61*.


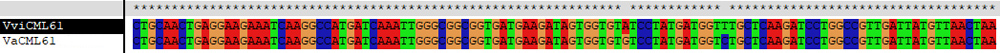


> VviCML61-VIT_205s0077g00300.1

ATGGAAAAGCTTGCATTTGTTGGACAGTTATGTTTCAGAAACAGAGAGAAGGCATGCTATTCCATCACAAGCACAACACCATATATATTGGCAGGTCTTGCTTCTGCCGTGGGTGGGTTGAGTGCACCATACGAAAAGGCTTCTCATGTACATGCAAGACCGATCGTGAATTGGCTCTGGTCAGCTGGTTGTCATCCGTTTGGACCATTCTCCAATACTTCCCAGATGAGTCAAATGCTCCAGGATGTTGCCTTGAGGAACACGATATACGCTCGTGTAGATTCTGCACTACACAGAATTCGGGATACATCAGAGTATGTACAAACTTTTGCTGCTGAATATCTGAAAACCCCACTTAGTGAACCAGTCAAGGGCAAGAAAAATAAGTCGAGCACTGAGCTATGGTTGGAAAAGTTCTACAAGAAGAAAACTAACTTGCCTGAACCTTTACCTCACGAATTAGTTGAAAGACTAGAGAAATTCTTGGATAATCACAACGGCTGCCCGGCCGAAAATCGTGTCAAAGATCTCCGCAGCTTTTACGTGGGATTCCCAAGCGGCGTCTCCGGAGATGAGGAGGCGATCGGGTCGATGATGTCGGTGGCGGATTCGAATAAGGATGGGTTTGTGGGGTACGATGAGTTTGAACACGTGTTGGGCTGCCGGAGAAGTCCGAGGAACAAGGGCCACGGCGTCGCTGGGGTGATGGAGGATGTGTGTAAGGTTATGGACAGGGACGGCGACGGCAAAGTAGGGCTCGAGGATTTGAAGAGCTATATGAATTGGGCCGGGTTTTCTGCAACTGAGGAAGAAATCAAGGCCATGATCAAATTGGGCGGCGGTGATGAAGATAGTGGTGTATCCTATGATGGTTTGCTCAAGATCCTGGCCGTTGATTATGTTAACTAA

>VviCML61-VIT_205s0077g00300.1

CTGCAACTGAGGAAGAAATCAAGGCCATGATCAAATTGGGCGGCGGTGATGAAGATAGTGGTGTATCCTATGATGGTTTGCTCAAGATCCTGGCCGTTGATTATGTTAACTAA

>VaCML61

CTGCAACTGAGGAAGAAATCAAGGCCATGATCAAATTGGGCGGCGGTGATGAAGATAGTGGTGTGTCCTATGATGGTCTGCTCAAGATCCTGGCCGTTGATTATGTTAACTAA
