# Supplementary material for: The Effect of Abiotic Stress Conditions on Expression of Calmodulin (CaM) and Calmodulin-Like (CML) Genes in Wild-Growing Grapevine Vitis amurensis
Source: Plants (Basel). 2019 Dec 13;8(12):602. doi: 10.3390/plants8120602 (PMC6963546; doi:10.3390/plants8120602)
Supplement: Supplementary file 1 [file plants-08-00602-s001.zip › plants-664661-supplementary/plants-664661-supplementary/Supplementary-664661/Supplementary-Table S1.docx]

**Supplementary**

**Table S1.** Primers used for amplification of full-length cDNA of calmodulin (*CaM*), calmodulin-like (*CML*) genes, and partial cDNA of house-keeping gene (*AtActin2*) in wild-growing grapevine *Vitis amurensis*.

| **cDNA (GeneBank)** | **Primer orientation** | **Primers, 5'-3'** |
| --- | --- | --- |
| *VaCaM8a* (MN515154) | F  R | ATGGCYGATCAGCTCACMGA  TCACTTCGCCATCATCACCT |
| *VaCam8b* (*MN515155)* | F  R | ATGGAGGATGTTCTCTTTCTC  TCACTTCGCCATCATCACCT |
| *VaCaM9* (MN478368) | F  R | ATGGCAGAACAGCTTACGGAA  TCACTTGGCAAGCATCATC |
| *VaCam10* (MN515156) | F  R | ATGGCCGATCAGCTCACTGA  TTAGAGGATGGTGCAGTGTG |
| *VaCML1* (MN537894) | F  R | ATGTCAACTCCTAGTTTTC  TTACATGGAGCGAGTCATCATG |
| *VaCML9a* (MN515159) | F  R | ATGAGACCGAACAACGGAGAT  TCAAACAGCTAACATCATTCTCAC |
| *VaCML9b* (MN515160) | F  R | ATGGCGAATGCACTGACAG  TTAATTGAAAGTCATCATCATCTT |
| *VaCML21* (MN540599) | F  R | ATGGGAGGCGTGGTGGG  TCAAACTTTCTCTTCACCTTC |
| *VaCML22* (MN540602) | F  R | ATGAAACAATCAGTGGGCAC  TTAACTTCCTGATATAGGAATTTCC |
| *VaCML41a* (MN537895) | F  R | ATGCAATTTCCTCCCACG  TTAAGCCATCATTTGATGAAAC |
| *VaCML41b* (MN537896) | F  R | ATGGCCACTGCTGCTG  TCAAGTCATCATCCGGTGAAA |
| *VaCML44* (MN537897) | F  R | ATGTCTTCGTCTTCCCTG  TCAGATGGTACTGAGGAG |
| *VaCML48* (MN562247) | F  R | ATGGCTTCCTTCTCCGG  CTACGCTACAAGAAATGGAATG |
| *VaCML51* (MN540594) | F  R | ATGTTTTCTGTGTTTCCCAT  TTACTTAATGCGATATCCTAGAG |
| *VaCML52* (MN540595) | F  R | ATGCCTCTGTGGACTCC  CTAACGTAACTTAAATCCCCAT |
| *VaCML53* (MN540596) | F  R | ATGCCTATGTGGAATCCC  CTAATTTGCGGGCAACC |
| *VaCML54* (MN540597) | F  R | ATGTGTGCCGTGGTGCC  TTACACTTTATAACCAAATTTCAG |
| *VaCML55* (MN540589) | F  R | ATGGCCATTAAGACGTATTA  CTAGAATTCCACGATCTTAACA |
| *VaCML57* (MN540598) | F  R | ATGCCTCGTMATTGGAA  TTATGCCTTGAYCKGATAT |
| *VaCML60* (MN537898) | F  R | ATGAAACTCATCTACAAGATCA  TCATGTCTGCTGCTCCATC |
| *VaCML62* (MN540605) | F  R | ATGAGTGTAGAAATTCTGAAT  TTACGGCACCAGTTTGGT |
| *VaCML65* (MN540606) | F  R | ATGAGTGTGGAAGTGTTG  TCAAGCATCCATTTTCATGG |
| *VaCML66* (MN540607) | F  R | ATGGGAGTGGTGATAATAG  TCAGGAAGAAGTCTTAGAGG |
| *VaCML71* (MN548771) | F  R | ATGGAAGTAGCCAAGAAG  TCAATGATGATTCTTGAGCTC |
| *VaCML72* (MN515163) | F  R | ATGGCGGAGCTTGAAGTTAA  TTATAACAAAGTAAGGCCTAAG |
| *VaCML73* (MN515164) | F  R | ATGGTTATGGATACACTGAA  CTAGAACACCGCAAGGCCA |
| *VaCML74* (MN537892) | F  R | ATGAGTGCAAGCGAACCT  TCAACCCCACAAATTGTCAAAAG |
| *VaCML75* (MN537893) | F  R | ATGAGCGTGGATGAACCGGT  CTAGGCCCATGAATTATCAAAAG |
| *VaCML76* (MN540613) | F  R | ATGGAGGTTGAGGGGGG  CTAAAACCATAGAGCTTCTTCAT |
| *VaCML77* (MN540608) | F  R | ATGTCTGGATATCCACACGC  CTAAGCAATAAGAAATGGCA |
| *VaCML78* (MN540610) | F  R | ATGGGGAAGGTGTCAGTA  TTAGAACTCATCATGGAAGTCG |
| *VaCML79* (MN515161) | F  R | ATGGCAGATGTGCTAAGC  CTAGGCGGTCATCATCATCT |
| *VaCML80* (MN515162) | F  R | ATGGGCAAAGATCTGAGC  TCACTTGGCCACCATCCG |
| *VviCML81* (MN540611) | F  R | ATGCCTTCTTTAACACCTCTG  TCAAGAAAGAAGTCCTGAAAT |
| *VaCML82* (MN540612) | F  R | ATGAGGATGAGAAGTCCC  TCAATTAAGAAAAATGTTGCCAG |
| *VaCML83* (MN540590) | F  R | ATGGAGAAGACACCAACAA  CTAAGAAAAACTTTTATCCAGAAACT |
| *VaCML84* (MN540591) | F  R | ATGTCCTTCTTCCAGTCCCA  TCAGATGAGGCAATTCTCTG |
| *VaCML85* (MN540592) | F  R | ATGGAGAAGACGACTCTTTTTC  TTAGCAGAAACTCTTCTCCACGA |
| *VaCML86* (MN540577) | F  R | ATGACGAGCAATTCTATTTC  TCACTGTTGACGCTGTTTAG |
| *VaCML87* (MN540593) | F  R | ATGGCGATTATGTGTTGC  TCACCAATTGTTCTCAGAAAT |
| *VaCML88* (MN540578) | F  R | ATGGAATCAGGTGGAGCC  TCAAGAGCTCCGAACAACTA |
| *VaCML89* (MN540579) | F  R | ATGTGCTACCAAATGCTC  CTATGATGAGGCTGAAAGAAG |
| *VaCML90-91* (MN540580) | F  R | ATGGATCCGGCAGAGCTG  CTATGAACCTGAACTACTCAAG |
| *VaCML92* (MN540581) | F  R | ATGAGAAGAGAAGATACAGCA  CTAGCGCATCATAGCGGA |
| *VaCML93* (MN540582) | F  R | ATGGCAAACGATGAGATGA  CTATAACATCATGACCTTGAACTC |
| *VaCML94* (MN540583) | F  R | ATGAAGAGAGATGTTCAATTTGAG  TTATAGCATCATAACTTTGAACTCAT |
| *VaCML95* (MN540584) | F  R | ATGATTAACTGCAGCATATA  TCAAAACATCATGAGCTTGAACTC |
| *VaCML96* (MN540585) |  |  |
| *VaCML100* (MN540586) |  |  |
| *VaCML103* (MN540587) | F  R | ATGATCAAYTGCRRYRTRTA  TYAMARCAWCATGAGCTTGA |
| *VaCML104* (MN540588) |  |  |
| *VaCML105* (MN562248) | F  R | ATGGCAAAGAATTCGTGTG  TTAAGAACGAGTCATCATCTT |
| *VaCML106* (MN562254) | F  R | ATGGCAGATAACCCCCAA  TTAAAAAACCTTAGCAACATCCTTC |
| *VaCML107* (MN562253) | F  R | ATGCAGTACCTGAGAGAGCT  CTAGTACCCATATGTAGTTCTCT |
| *VaCML108* (MN562252) | F  R | ATGACTGAGTTCAAG  CTAGTACCCATATGTAGTTCTCT |
| *VaCML109* (MN562249) | F  R | ATGAAACTCATCTACAAGATCA  TCATGTCTGCTGCTCCATC |
| *VaCML110* (MN562246) | F  R | ATGCCAACCTTTTTGCATAG  CTAACTCAATGCACTGAAACC |
| *AtActin2* (NM_112764) | F  R | GAT TCA GAT GCC CAG AAG TC  TCT GTG AAC GAT TCC TGG A |
