# Supplementary material for: The Effect of Abiotic Stress Conditions on Expression of Calmodulin (CaM) and Calmodulin-Like (CML) Genes in Wild-Growing Grapevine Vitis amurensis
Source: Plants (Basel). 2019 Dec 13;8(12):602. doi: 10.3390/plants8120602 (PMC6963546; doi:10.3390/plants8120602)
Supplement: Supplementary file 1 [file plants-08-00602-s001.zip › plants-664661-supplementary/plants-664661-supplementary/Supplementary-664661/Supplementary-Table S2.docx]

**Supplementary**

**Table S2.** Primers used for real-time PCR for calmodulin (*CaM*) and calmodulin-like (*CML*) genes in wild-growing grapevine *Vitis amurensis*.

| **cDNA (GeneBank)** | **Primer orientation** | **Primers, 5'-3'** |
| --- | --- | --- |
| *VaCaM8a* (MN515154) | F  R | GATGATTCGGGAGGCTGATG TCACTTCGCCATCATCACCT |
| *VaCam8b* (*MN515155)* | F  R | GATGATTCGGGAGGCTGATG TCACTTCGCCATCATCACCT |
| *VaCaM9* (MN478368) | F  R | GGTGGATGAGATGATTCGAGA TCACTTGGCAAGCATCATC |
| *VaCam10* (MN515156) | F  R | ATGGCCGATCAGCTCACTGA  TTAGAGGATGGTGCAGTGTG |
| *VaCML1* (MN537894) | F  R | TGTTGGAGGTGTTGGGGAG  TTACATGGAGCGAGTCATCATG |
| *VaCML9a* (MN515159) | F  R | TGGTTACATATCAGCTAATGAAT TCAAACAGCTAACATCATTCTCAC |
| *VaCML9b* (MN515160) | F  R | AGAGGCTGATATGGATGGTGAT  TTAATTGAAAGTCATCATCATCTT |
| *VaCML21* (MN540599) | F  R | ATGGGAGGCGTGGTGGG  ACCATTTTGGCCTCAAGC |
| *VaCML22* (MN540602) | F  R | TAATGGAACCTTCCACCTCT  AGCTGTGGTGAACCCATC |
| *VaCML41a* (MN537895) | F  R | ATGCAATTTCCTCCCACG  GCCTATGGAGACTTAACCT |
| *VaCML41b* (MN537896) | F  R | ATGGCCACTGCTGCTG  CGAGGGAGGCTGAGTTTA |
| *VaCML44* (MN537897) | F  R | TGTATGACACCAACTCTGAC  TCAGATGGTACTGAGGAG |
| *VaCML48* (MN562247) | F  R | GATCCACGTTACACTGGTTCA  CTACGCTACAAGAAATGGAATG |
| *VaCML51* (MN540594) | F  R | CAACTCCGAGGAGATGG  TTACTTAATGCGATATCCTAGAG |
| *VaCML52* (MN540595) | F  R | GGAGACCATTTCATTAGCGA  CTAACGTAACTTAAATCCCCAT |
| *VaCML53* (MN540596) | F  R | GACCGATACATTAGCAAGGA  CTAATTTGCGGGCAACC |
| *VaCML54* (MN540597) | F  R | GGAGTTGACCAGTCTCGT  TTACACTTTATAACCAAATTTCAG |
| *VaCML55* (MN540589) | F  R | GATGTGAATGGCGACGG  CTAGAATTCCACGATCTTAACA |
| *VaCML57* (MN540598) | F  R | TGGGTTTAGAGCATGGAGAG  CTCCTGCTGGCTGATGTG |
| *VaCML60* (MN537898) | F  R | CGGTGCACGTTAGAGGAT  TCATGTCTGCTGCTCCATC |
| *VaCML62* (MN540605) | F  R | GGGTTAGGGCTCTTGCCTT  TTACGGCACCAGTTTGGT |
| *VaCML65* (MN540606) | F  R | GCTTCCTGAAGAAAGCTGTT  TCAAGCATCCATTTTCATGG |
| *VaCML66* (MN540607) | F  R | GGCTCTTGAGGACGATG  TCAGGAAGAAGTCTTAGAGG |
| *VaCML71* (MN548771) | F  R | GTTGATGGAGGAGTCTGAG  TCAATGATGATTCTTGAGCTC |
| *VaCML72* (MN515163) | F  R | TCCGGGAGGCTGATACAAACG  TTATAACAAAGTAAGGCCTAAG |
| *VaCML73* (MN515164) | F  R | GTCATATCGAGAACTCTCTGATA  CTAGAACACCGCAAGGCCA |
| *VaCML74* (MN537892) | F  R | ACCGACGGTGATGGCTGCAT  TCAACCCCACAAATTGTCAAAAG |
| *VaCML75* (MN537893) | F  R | AGGCAGACACTGACGGCGAT  CTAGGCCCATGAATTATCAAAAG |
| *VaCML76* (MN540613) | F  R | GCAGTGGGTGGAGGAAAT  CTAAAACCATAGAGCTTCTTCAT |
| *VaCML77* (MN540608) | F  R | CTTTCCAAGTTTGATAAGAGTGG  TTCAATAGCCTTGTTTCTGC |
| *VaCML78* (MN540610) | F  R | CGTCTAACCTTGACAGAGA  TTAGAACTCATCATGGAAGTCG |
| *VaCML79* (MN515161) | F  R | CAGAGAGGCCGACTTGGA  CTAGGCGGTCATCATCATCT |
| *VaCML80* (MN515162) | F  R | TCGCCGATCTCCGCCACA  TCACTTGGCCACCATCCG |
| *VviCML81* (MN540611) | F  R | CGATGATGGAAATTTAAGCTCAG  TCAAGAAAGAAGTCCTGAAAT |
| *VaCML82* (MN540612) | F  R | AGCCAGGGTTCATGGGATTGC  TCAATTAAGAAAAATGTTGCCAG |
| *VaCML83* (MN540590) | F  R | TCACAAGTAGAGGACTGCAGAAGG  CTAAGAAAAACTTTTATCCAGAAACT |
| *VaCML84* (MN540591) | F  R | TGGAAGGATTGATTTCAATGAATTTGT  TCAGATGAGGCAATTCTCTG |
| *VaCML85* (MN540592) | F  R | TGGAATGCGAGAAAATGATTCAAG  TTAGCAGAAACTCTTCTCCACGA |
| *VaCML86l* (MN540577) | F  R | GGATCTTTTGACTCCGACG  TCACTGTTGACGCTGTTTAG |
| *VaCML87* (MN540593) | F  R | CCAAGGAAATGGAAAAGCT  TCACCAATTGTTCTCAGAAAT |
| *VaCML88* (MN540578) | F  R | ACCAGGACGGGCGCGT  TCAAGAGCTCCGAACAACTA |
| *VaCML89* (MN540579) | F  R | AGATGATAAGGAAGGTAGACA  CTATGATGAGGCTGAAAGAAG |
| *VaCML90-91* (MN540580) | F  R | ACAGAAGGTGGATGTTGATGG  CTATGAACCTGAACTACTCAAG |
| *VaCML92* (MN540581) | F  R | AACGGAGATGGCGTCCTC  CTAGCGCATCATAGCGGA |
| *VaCML93* (MN540582) | F  R | GTTGCATCACCCCAAAGAGCTTG  ATCGATGCTCCGGGATTCACCA |
| *VaCML94* (MN540583) | F  R | GGTGGATGAATGCAGGGT  TTATAGCATCATAACTTTGAACTCAT |
| *VaCML95* (MN540584) | F  R | CAGTGTTATGATCAGGCAGTTTG  TCAAAACATCATGAGCTTGAACTC |
| *VaCML96* (MN540585) |  |  |
| *VaCML100* (MN540586) |  |  |
| *VaCML103* (MN540587) |  |  |
| *VaCML104* (MN540588) |  |  |
| *VaCML105* (MN562248) | F  R | CGAGAAGGTCACCCTCAA  TTAAGAACGAGTCATCATCTT |
| *VaCML106* (MN562254) | F  R | ATTTGCAGACTTCCACCGTG  TTAAAAAACCTTAGCAACATCCTTC |
| *VaCML107* (MN562253) | F  R | ATGCAGTACCTGAGAGAGCT  CTGAAGAGTCCTGAAAGAATTTAG |
| *VaCML108* (MN562252) | F  R | ATGACTGAGTTCAAG  CTTCTTTTATCTCTTGCTTCTTCTG |
| *VaCML109* (MN562249) | F  R | CGGTGCACGTTAGAGGAT  TCATGTCTGCTGCTCCATC |
| *VaCML110* (MN562246) | F  R | CTGCAAGAGAATGATAATGAAGG  CTAACTCAATGCACTGAAACC |
