# Supplementary figures and images for: The Effect of Abiotic Stress Conditions on Expression of Calmodulin (CaM) and Calmodulin-Like (CML) Genes in Wild-Growing Grapevine Vitis amurensis
Source: Plants (Basel). 2019 Dec 13;8(12):602. doi: 10.3390/plants8120602 (PMC6963546; doi:10.3390/plants8120602)

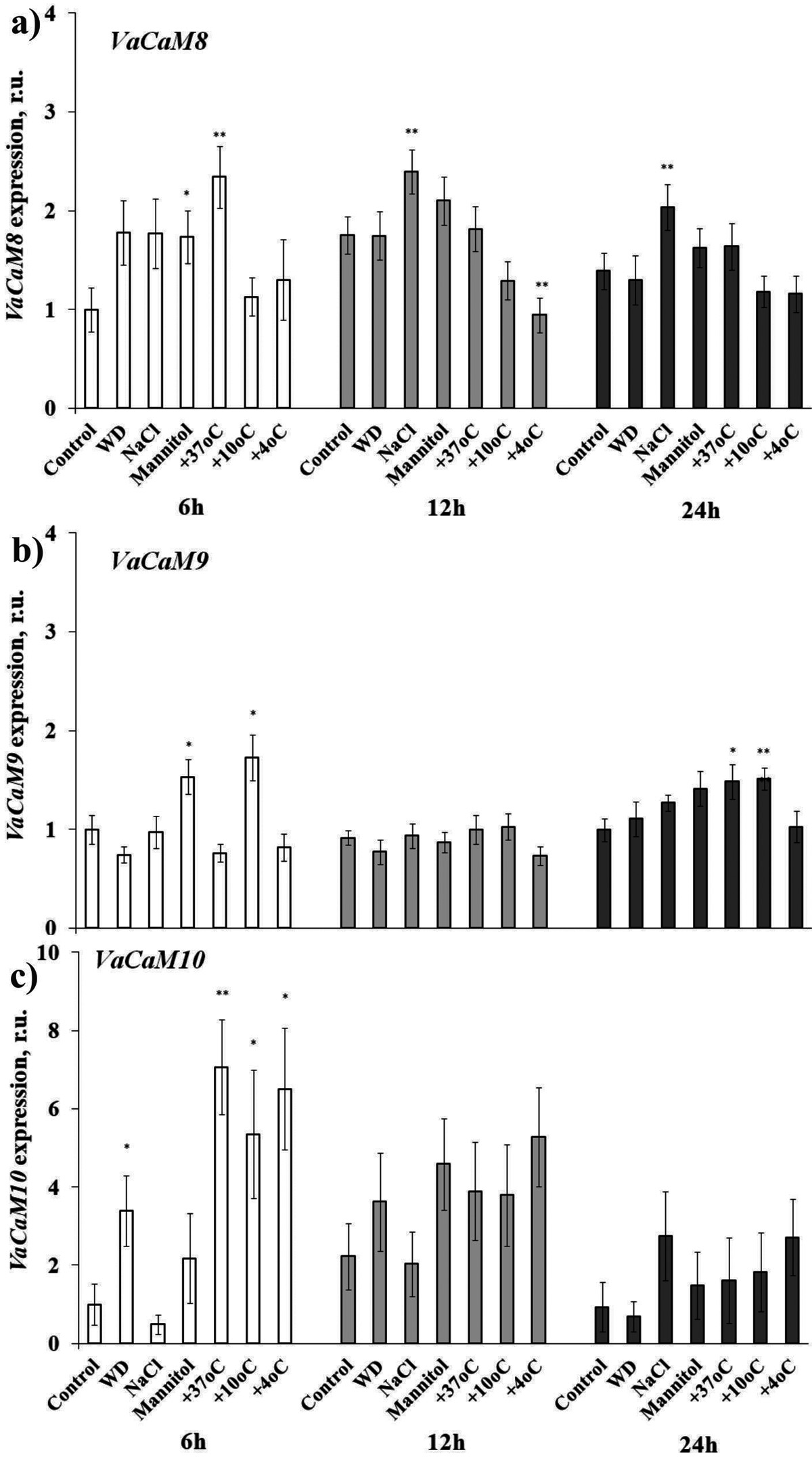

Supplement: Supplementary file 1 [file plants-08-00602-s001.zip › plants-664661-supplementary/plants-664661-supplementary/Supplementary-664661/Fig.S1.jpg]

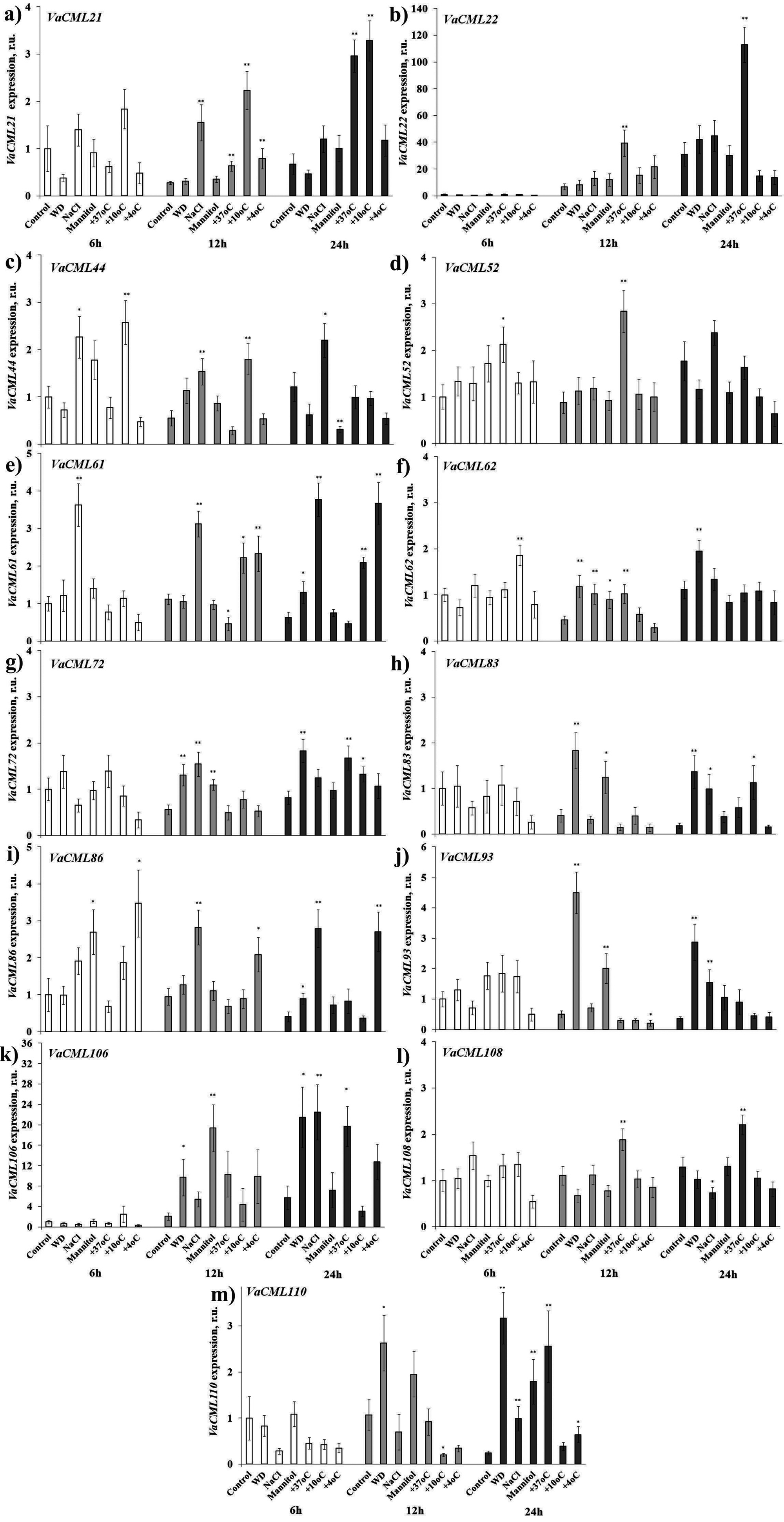

Supplement: Supplementary file 1 [file plants-08-00602-s001.zip › plants-664661-supplementary/plants-664661-supplementary/Supplementary-664661/Fig.S2.jpg]

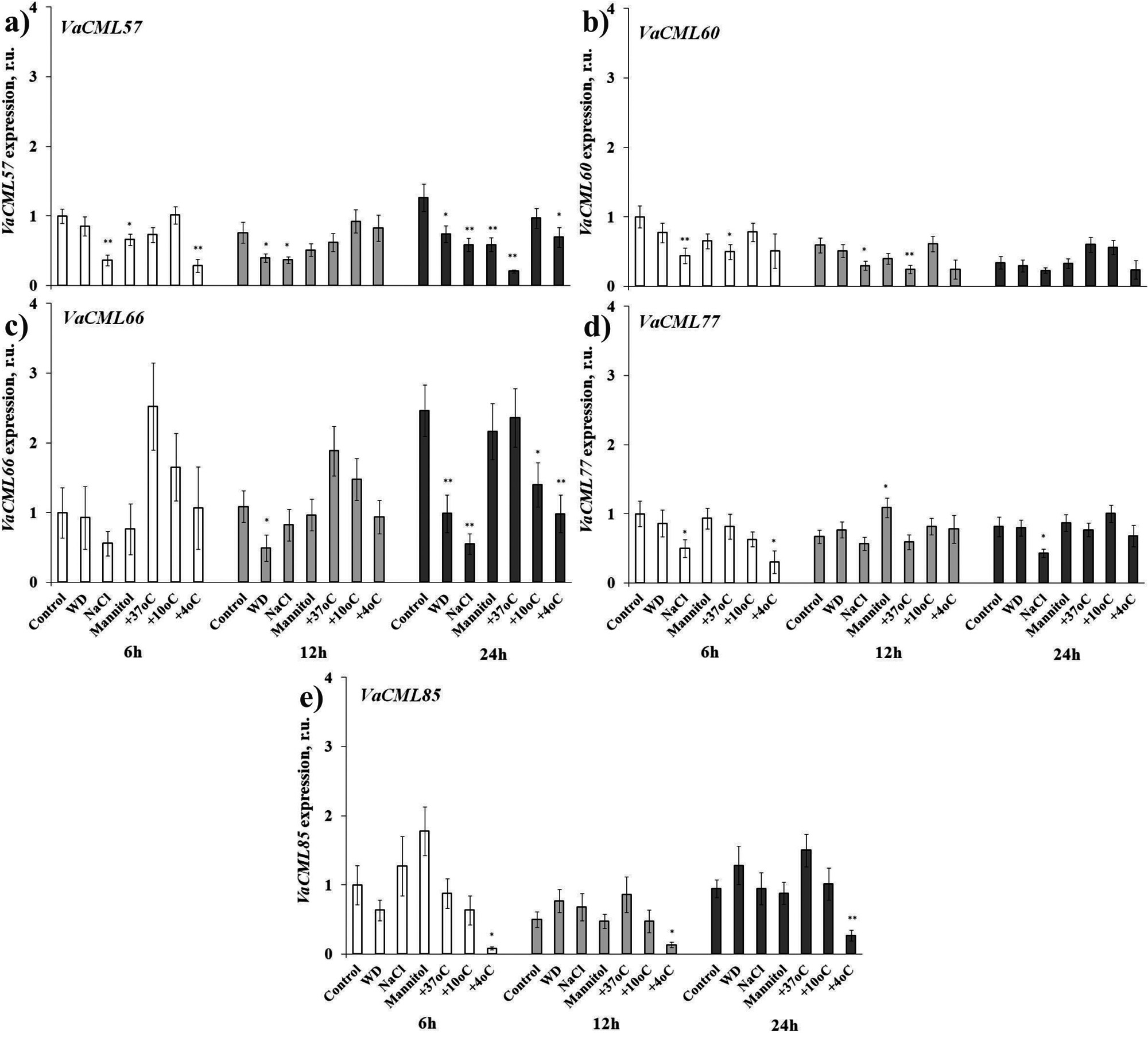

Supplement: Supplementary file 1 [file plants-08-00602-s001.zip › plants-664661-supplementary/plants-664661-supplementary/Supplementary-664661/Fig.S3.jpg]

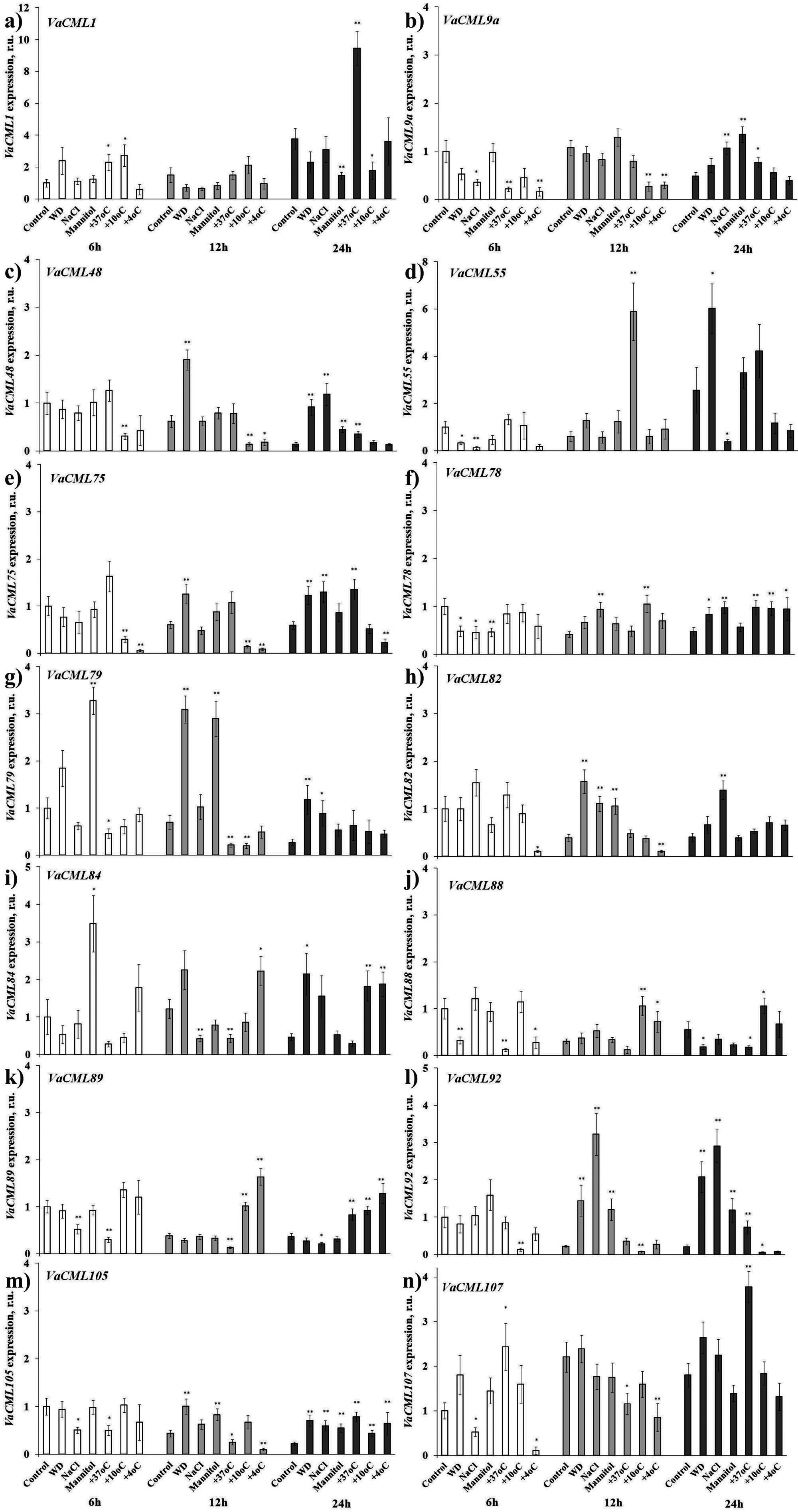

Supplement: Supplementary file 1 [file plants-08-00602-s001.zip › plants-664661-supplementary/plants-664661-supplementary/Supplementary-664661/Fig.S4.jpg]

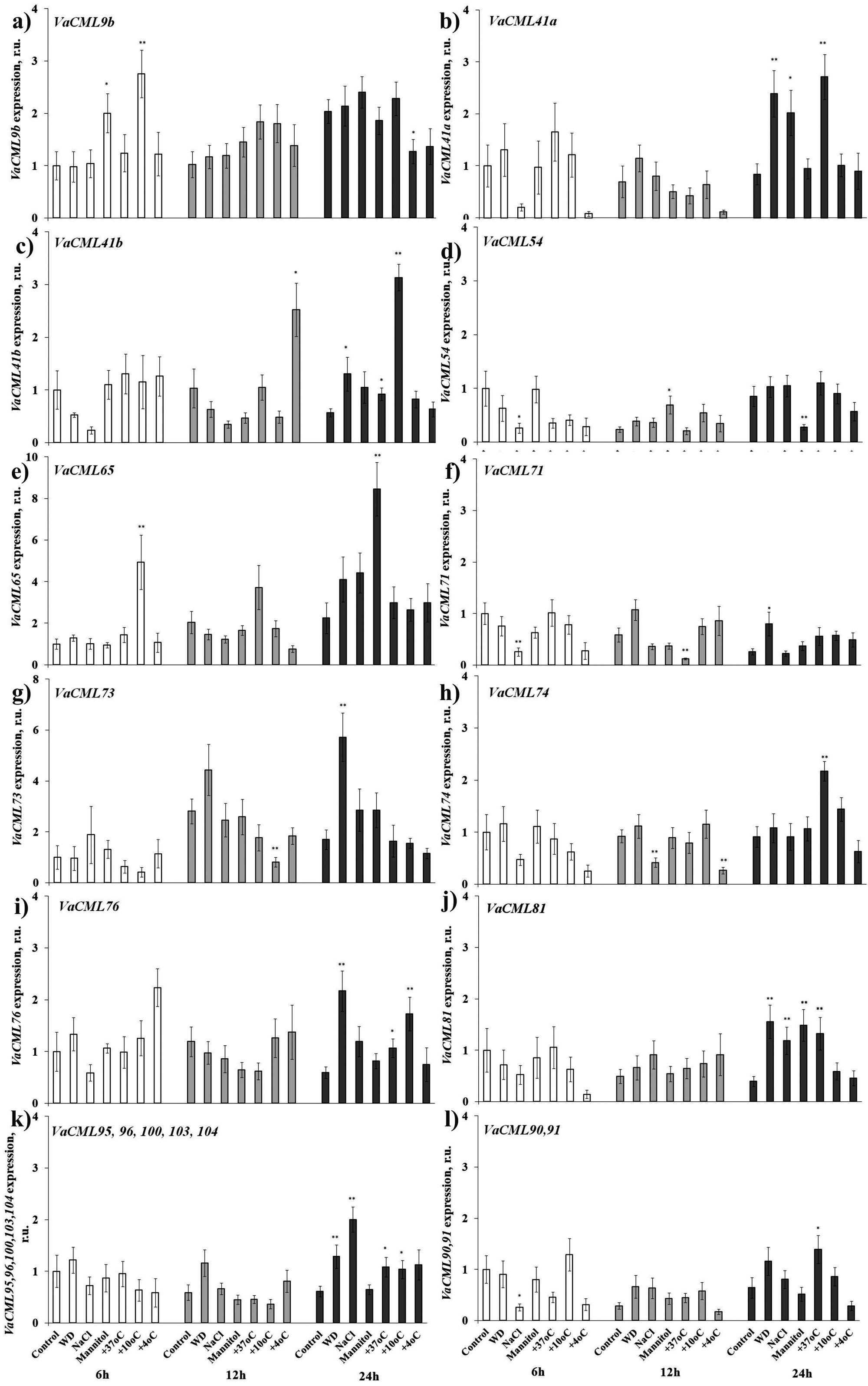

Supplement: Supplementary file 1 [file plants-08-00602-s001.zip › plants-664661-supplementary/plants-664661-supplementary/Supplementary-664661/Fig.S5.jpg]

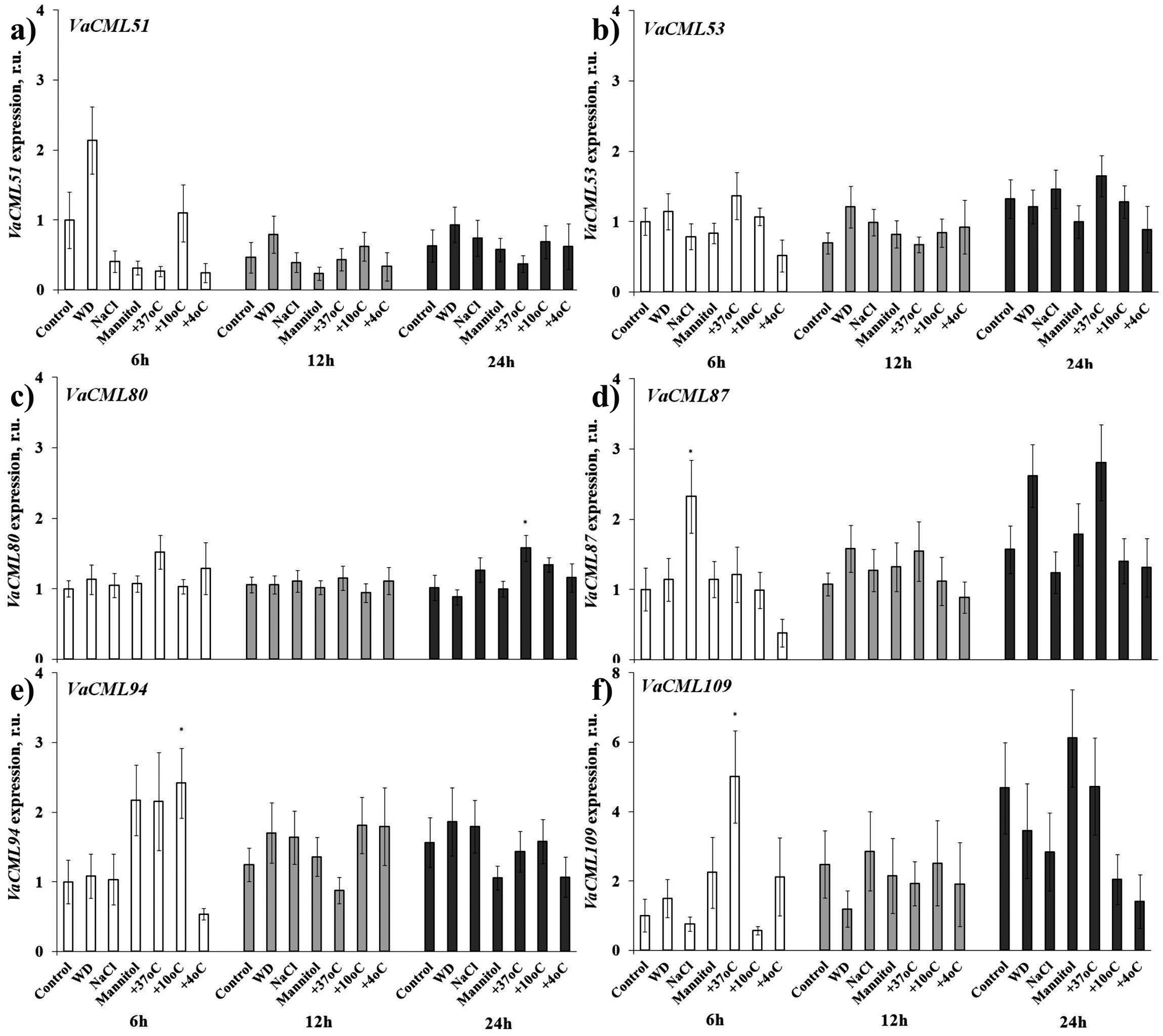

Supplement: Supplementary file 1 [file plants-08-00602-s001.zip › plants-664661-supplementary/plants-664661-supplementary/Supplementary-664661/Fig.S6.jpg]

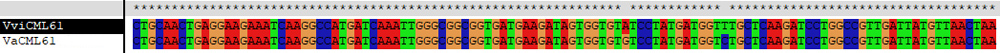

Supplement: Supplementary file 1 [file plants-08-00602-s001.zip › plants-664661-supplementary/plants-664661-supplementary/Supplementary-664661/Fig.S7.jpg]
